# Selective Electrochemical Reduction of Nitrogen to Ammonia by Adjusting the Three-Phase Interface

**DOI:** 10.34133/2019/1401209

**Published:** 2019-11-30

**Authors:** Haiyan Wang, Yuzhuo Chen, Ruxue Fan, Jiadong Chen, Zhe Wang, Shanjun Mao, Yong Wang

**Affiliations:** Advanced Materials and Catalysis Group, Institute of Catalysis, Department of Chemistry, Zhejiang University, Hangzhou 310028, China

## Abstract

The electrochemical nitrogen reduction reaction (NRR) provides a sustainable and alternative avenue to the Haber-Bosch process for ammonia (NH_3_) synthesis. Despite the great efforts made on catalysts and electrolytes, unfortunately, current NRR suffers from low selectivity due to the overwhelming competition with the hydrogen evolution reaction (HER). Here, we present an adjusted three-phase interface to enhance nitrogen (N_2_) coverage on a catalyst surface and achieve a record-high Faradic efficiency (FE) up to 97% in aqueous solution. The almost entirely suppressed HER process combined with the enhanced NRR activity, benefiting from the efficient three-interface contact line, is responsible for the excellent selectivity toward NH_3_, as evidenced by the theoretical and experimental results. Our strategy also demonstrates the applicability to other catalysts that feature strong H adsorption ability, to boost the FE for NH_3_ synthesis above 90% and to improve the NRR activity by engineering the catalysts.

## 1. Introduction

The electrochemical nitrogen reduction reaction (NRR) to ammonia (NH_3_) at ambient conditions provides a sustainable and distributed alternative to the Haber-Bosch process for making fertilizers and energy carriers [1–5]. However, the inertness of nitrogen (N_2_) with a strong N≡N triple bond (bonding energy of 940.95 kJ mol^−1^), nonpolarity, and negative electron affinity typically result in a large overpotential and a very low Faradic efficiency (FE) (typically <10%) for NRR [6–8]. In addition, the NRR competes fiercely with the hydrogen evolution reaction (HER). Most metal catalysts with low NRR overpotentials favor adsorption of H over N_2_, resulting in a tendency to produce hydrogen (H_2_) rather than NH_3_ [9–11]. Furthermore, the large energy gap (10.82 eV) between the highest occupied and the lowest unoccupied molecular orbitals of N_2_ does not favor the one- or two-electron transfer process and therefore endows the NRR with sluggish kinetics [12–14]; most protons and electrons go toward generating H_2_. Consequently, the selectivity remains a major challenge for the multielectron and multiproton NRR pathways in competing with the dominant HER for active catalysts.

Substantial efforts have been dedicated to optimization of the NRR selectivity by developing various heterogeneous electrocatalysts including metals, metal oxides, and nonmetallic materials, particularly focused on tailoring the particle size, crystallinity, morphology, defect density, and surface active sites [15–28]. For example, single-atom catalysts were demonstrated to have an atomic ensemble effect on suppressing the HER since it was perceived that only the top site of single-atom catalysts with positive relative energies contributed to H adsorption, with respect to the multiple adsorption sites (top, bridge, and hollow) for bulk metal surfaces [29]. But to date, the highest FE achieved for these single-atom catalysts is limited to 56.6% as the HER remains to be a highly competitive reaction [30]. Recently, the rates of NH_3_ and H_2_ production were deemed to be, respectively, zeroth order and first order in the electron and proton concentrations [31]. Thus, it was suggested that reducing the proton and electron supply would slow down the HER kinetics and make N_2_ more accessible to the surface active sites, thereby promoting the NRR conversion. In this respect, a number of strategies were implemented to expect an improvement on the selectivity toward NH_3_ by using an aprotic solvent with extremely few proton donors [32, 33], a hydrophobic protection layer to hinder proton transport [33, 34], or an insulator-metal-catalyst electrode to slow down the electron tunneling [16, 31]. Yet in spite of the small increase in selectivity, the NH_3_ yield decreased significantly due to the proton-coupled electron transfer reaction mechanism [35]. Conventionally, a gas reactant needs to dissolve in the electrolyte solution and electrochemical reactions take place in the solid-liquid two-phase interface [36]. Thus, the electrocatalytic activity was limited by the inefficient gas mass transport seriously. Accordingly, another method for preferential production of NH_3_ is to increase the concentration of N_2_ in electrolyte solution in view of the fact that most organic solvents can dissolve N_2_ by one or two orders of magnitude higher than water. An impressive FE of 60% was achieved by adopting an aprotic fluorinated ionic liquid which had higher N_2_ solubility compared with water (17 vs. 0.66 mmol L^−1^) [37]. Nevertheless, the NH_3_ production rate was limited to 10^−12^ mol s^−1^ cm^−2^. A recent work achieved high NRR selectivity (90%) via a superhydrophobic MOF layer to repel water molecules and trap N_2_, but at a cost of low activity [34]. Hence, the accessibility of adequate N_2_ molecules to the catalyst is critical but generally deficient, and it is difficult to obtain orders of magnitude improvement in selectivity while maintaining the activity by only manipulating the concentration of N_2_ [31], although a theoretical study has pointed out the preference for NH_3_ formation if the catalyst surface is covered with N_2_ rather than H adatoms [10]. Previous reports have found that the selectivity and activity of CO_2_ reduction are significantly affected by the CO_2_ concentration at the catalyst surface and both can be improved by adjusting the reaction interface [38, 39]. Besides, the solid-state electrochemical NRR cell with electrodes exposed to N_2_ also demonstrates the promise to overcome the low conversion of the conventional catalytic reactors, but its performance is limited by the relative poor transport of protons and electronics [40]. Therefore, we believe that effective electrochemical reduction of nitrogen to ammonia requires efficient contact of N_2_ (gas), H^+^ (liquid), and catalyst (solid).

In the present work, we proposed a simple strategy to enhance the coverage of N_2_ on the catalyst surface in aqueous acid and promote the gas-solid catalyst-liquid electrolyte three-phase reaction interface. Different from the traditional reaction interface, in the adjusted three-phase interface, N_2_ does not have to be dissolved in the electrolyte and N_2_ bubbles surrounded the catalyst to promote substantial N_2_ molecules adsorbing and occupying the active sites preferentially. A high FE to NH_3_ of ~97% was achieved on a common supported Pd catalyst at 0.1 V vs. the reversible hydrogen electrode (RHE) in an acidic aqueous solution (0.1 M HCl). Our strategy could be easily extended to other catalysts such as Ir and RuPd, though they feature strong H adsorption ability to boost the FE for electrocatalytic NRR. Moreover, excellent NRR activity was then achieved by employing the adjusted three-phase interface and engineering catalysts with more active sites. Our results offer a new outlook to break through the limitations in selectivity toward NH_3_ from renewable energy sources especially in aqueous solution and also provide valuable insights to enhance the electrocatalytic performance of solid catalysts in a proton-sensitive competition system.

## 2. Results

### 2.1. Theoretical Insights into the Suppression of HER

**Figure 1:**
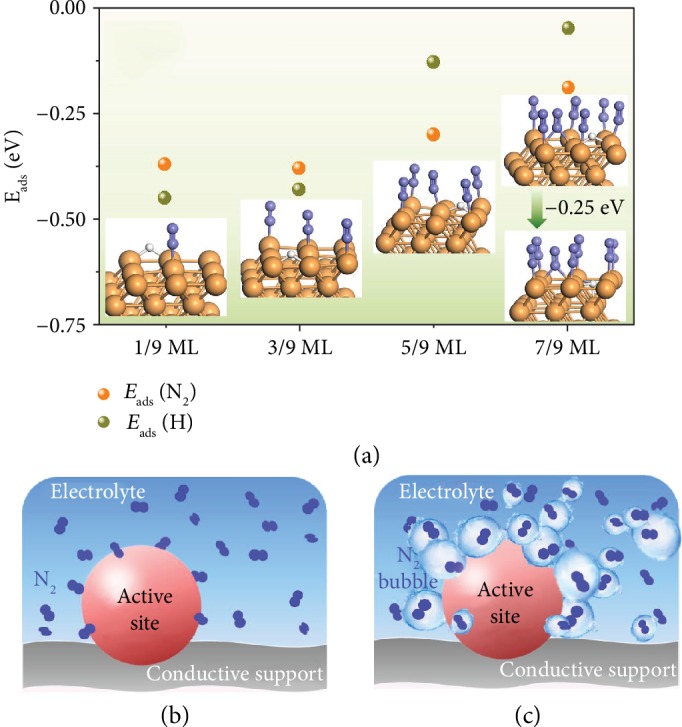
Computational studies and schematics of the conventional and adjusted reaction interface. (a) DFT calculations on the effect of *θ*_N_2__ on *E*_ads_(H) on the Pd(100) surface. White and purple balls denote H and N, respectively. (b) Schematic of the traditional solid-solution two-phase interface. (c) Schematic of the adjusted gas-solid-solution three-phase interface.

Increasing N_2_ coverage (*θ*_N_2__) on the electrocatalyst should be an effective strategy to increase the N_2_ selectivity to NH_3_, and the HER process can be suppressed seriously (see Supplemental Information: Effect of *θ*_N_2__ on selectivity). In order to gain a theoretical insight into the suppression of HER with the increase in *θ*_N_2__ on the Pd surface, a *p*(3 × 3) model for Pd(111), Pd(100), and Pd(211) facets with various *θ*_N_2__ was built and the following H adsorption has been investigated using density functional theory (DFT) calculations in this work. The adsorption configuration of N_2_ was chosen to be vertical rather than parallel ([Supplementary-material supplementary-material-1]), which means *θ*_N_2__ can vary from 1/9 ML to 1 ML on Pd(111) and Pd(100) facets and 1/9 ML to 6/9 ML on Pd(211) theoretically owing to its step-like property and the repulsion between vertical adsorbed N_2_. As shown in Figure 1(a), *E*_ads_(H) decreases with the increase in *θ*_N_2__ on the Pd(100) facet and quickly approaches 0 at 7/9 ML of *θ*_N_2__, implying the gradually weakened adsorption of H. Additionally, H prefers permeating into Pd bulk, forming a PdH compound, rather than being on the surface stably at high *θ*_N_2__ such as 7/9 ML (Figure 1(a)). As a result, the HER process, which requires surface-adsorbed H on the Pd(100) surface, was extensively suppressed. Similar phenomena could occur on other facets such as Pd(211) and Pd(111) (Figures [Supplementary-material supplementary-material-1]). Hence, one can conclude that the HER process can be greatly repressed with the increase in *θ*_N_2__ on the Pd surface via inhibiting the stable adsorption of H on active sites, thereby enhancing N_2_ reduction selectivity toward NH_3_. However, traditionally, N_2_ is dissolved in the electrolyte and the NRR occurs in the solid-liquid two-phase interface, solid catalyst-electrolyte solution, as shown in Figure 1(b). As the solubility of N_2_ in water is as low as 0.66 mmol L^−1^ under standard temperature and pressure, corresponding to ~5 orders of magnitude fewer N_2_ molecules than water molecules, the NRR activity is severely limited by the inefficient N_2_ mass transport and H (or H_2_O) has the priority over N_2_ to occupy the active sites, leading to a dominant HER process and poor selectivity to NH_3_. Therefore, in order to increase the coverage of N_2_ instead of H adatoms on the catalyst surface, the gas- (N_2_) solid- (catalyst) liquid (electrolyte solution) three-phase contact line should be enhanced to promote efficient gas mass transport. In the adjusted three-phase interface (Figure 1(c)), contrary to the traditional reaction interface (Figure 1(b)), N_2_ does not have to be dissolved in the electrolyte and the catalysts are surrounded by N_2_ bubbles, and thereby adequate N_2_ molecules can adsorb and occupy the active sites preferentially, consequently hindering the stable adsorption of H and suppressing the HER. With N_2_ bubbles covering catalyst surfaces, NRR can take place at the interface where N_2_ bubbles, solid catalysts, and electrolytes contact meanwhile, thus improving the electrocatalytic NRR performances.

### 2.2. Catalyst Synthesis and Characterization

**Figure 2:**
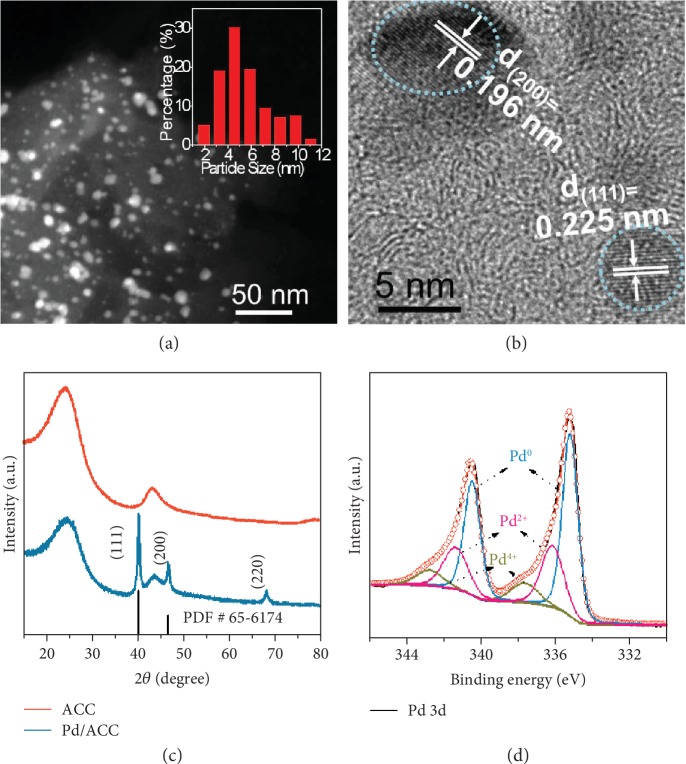
Structure analyses of the Pd/ACC catalyst. (a) Representative HAADF-STEM image of the Pd/ACC catalyst with an inset of particle size distribution. (b) HRTEM image of the Pd/ACC catalyst. (c) XRD images of the ACC and Pd/ACC catalysts. (d) Pd 3d XPS spectrum of the Pd/ACC catalyst.

To demonstrate the feasibility of the adjusted three-phase interface for electrochemical NRR, a common Pd/activated carbon cloth (Pd/ACC) catalyst was prepared via an ultrasound-assisted reduction technique. The ACC support had a microporous structure and a specific surface area of 96 m^2^ g^−1^ ([Supplementary-material supplementary-material-1]). The representative high-angle annular dark-field scanning transmission electron microscopy (HADDF-STEM) image revealed well-dispersed nanoparticles with an average size centered at 4.5 nm (Figure 2(a)). The lattice spacing of the nanoparticles was measured to be 0.196 nm and 0.225 nm, corresponding to the (200) and (111) planes of Pd, respectively (Figure 2(b)). The X-ray diffraction (XRD) pattern of Pd/ACC could be indexed to face-centered cubic (fcc) Pd (PDF#65-6174) (Figure 2(c)). Additionally, X-ray photoelectron spectroscopy (XPS) results showed the presence of C, O, and Pd in the catalyst without the signal of N ([Supplementary-material supplementary-material-1]), and the surface Pd was mainly in a metallic state and partially oxidized to Pd^2+^ and Pd^4+^ (Figure 2(d)).

### 2.3. Electrocatalytic Performances

**Figure 3:**
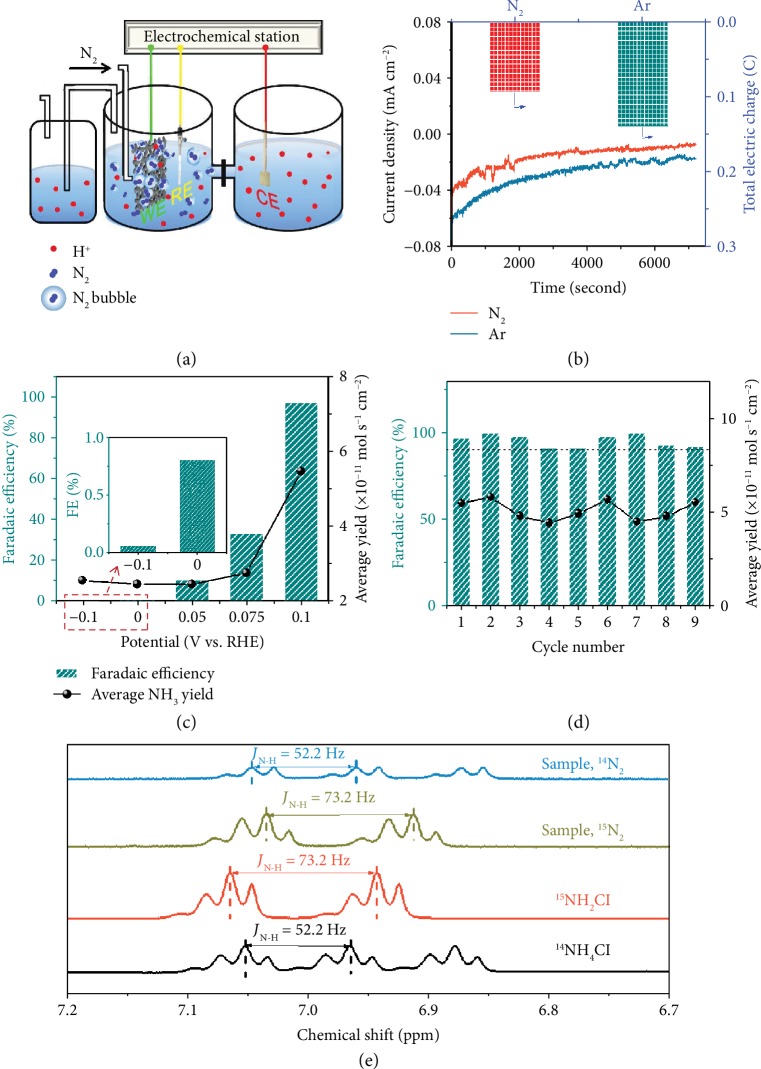
Electrochemical NRR performances of the Pd/ACC catalyst. (a) Schematic of the NRR device. (b) Chronoamperometry curve and total electric charge after electrolysis of Pd/ACC in N_2_ or Ar at 0.1 V for 2 h. (c) FE and average yield rate of NH_3_ production at various potentials based on the indophenol blue method with an inset of an enlarged figure. (d) Nine times recycling NRR experiments of a Pd/ACC electrode tested at 0.1 V for 2 h. (e) Isotope labelling experiment. 600 M ^1^H NMR spectra were obtained after electrolysis in 0.1 M HCl with ^15^N_2_ and ^14^N_2_ as the feeding gas. The multiplet splitting of the peaks may because of the deuterated derivatives of ammonium.

The electrocatalytic NRR activities of the Pd/ACC catalyst were then measured in 0.1 M HCl solution in a gas-tight two-compartment electrochemical cell separated with a Nafion 211 membrane and connected with a gas absorber (Figure 3(a)). To reduce a false positive from potential contaminant in the input gas stream, ultra-high-purity N_2_ (99.9999% purity) was used as the feeding gas and the potential NO*_x_* in N_2_ was detected with a nitrite assay kit and by gas chromatography and diffuse reflectance infrared Fourier transform spectroscopy (DRIFTS) before NRR tests (see Supplemental Information and Determination of NO*_x_* Contamination in Ultra-High-Purity N_2_ Gas). Almost no nitrite was detected after N_2_ flowing for 2 h (~1 ppb, [Supplementary-material supplementary-material-1]), indicating that the NO*_x_* in ultra-high-purity N_2_ could be ignored. Gas chromatographic curve ([Supplementary-material supplementary-material-1]) and DRIFTS spectrum ([Supplementary-material supplementary-material-1]) both show no signal of NO*_x_* which corresponds well with the nitrite assay kit result, further demonstrating the high purity of feeding gas. Then, N_2_ gas was supplied in a continuous feed stream with a flow rate of 130 sccm to the cathode compartment. The gas tube was positioned near the electrode to ensure that the entire Pd/ACC catalyst was surrounded by a mass of N_2_ bubbles during the electrocatalytic process ([Supplementary-material supplementary-material-1]), expecting to adjust the gas-solid-liquid three-phase contact interface and increase the coverage of N_2_ on the catalyst surface.

In order to verify the feasibility of this strategy, linear sweep voltammetry (LSV) was conducted on Pd/ACC in N_2_-saturated and Ar-saturated HCl solution with bubbles covering the catalyst in the same cell. As shown in [Supplementary-material supplementary-material-1], the reduction current density of N_2_ bubbles covering Pd/ACC is slightly smaller than that of Ar bubbles, implying that some nitrogen species may be adsorbed on the catalysts hindering the HER activity when Ar was changed to N_2_ [41]. In addition, once N_2_ flow (red curve in [Supplementary-material supplementary-material-1]) was changed to Ar with the same cell assembly, the current density increased immediately, reconfirming that the decreased current density was due to the impeditive HER in the N_2_-catalyst-electrolyte three-phase interface. These phenomena were repeated and became more obvious with chronoamperometry. Catalysts surrounded by N_2_ bubbles might have two mechanisms in impeding H_2_ production: (1) reducing the proton donors with bubbles and (2) occupying more active sites of the catalyst with nitrogen. To gain further insight into the suppressed HER, Ar gas was injected into the cathode compartment and LSV was tested on Pd/ACC with or without Ar bubbles covering by adjusting the position of the gas tube. As shown in [Supplementary-material supplementary-material-1], the LSV curves coincide well with each other, suggesting that the inert Ar bubbles have no discernible effect on H_2_ production in the three-phase interface, and therefore, one could conclude that nitrogen adsorbed on the active sites is the main reason for the inhibited HER under the adjusted three-phase interface, which corresponds well with the DFT results. Based on the above, we believe that the possible mechanism of this three-phase boundary control is that once the catalyst sites are exposed to the nitrogen gas (via bubbles), the hydrogen is replaced by it and NRR can take place at the interface where N_2_ bubbles, solid catalysts, and electrolytes contact meanwhile.

Subsequently, to further study the impact of this adjusted three-phase interface, chronoamperometry was tested on Pd/ACC and the electrochemical NRR performances were systematically investigated. Unless otherwise specified, all the potentials were converted and reported as values vs. RHE. As shown in Figure 3(b), Pd/ACC with N_2_ bubbles produces a smaller current density after continuous electrolysis at 0.1 V for 2 h than that with Ar bubbles, which demonstrates that the competing HER was suppressed during the NRR process obviously. These results correspond well with those obtained from the above LSVs. The primary NRR product NH_3_ was further quantified by the indophenol blue ([Supplementary-material supplementary-material-1]) and salicylic acid methods ([Supplementary-material supplementary-material-1]) simultaneously, while the yield of the by-product N_2_H_4_ was determined by the para-dimethylaminobenzaldehyde method ([Supplementary-material supplementary-material-1]). In our system, only NH_3_ was detected without the presence of N_2_H_4_ ([Supplementary-material supplementary-material-1]), implying good selectivity for N_2_ reduction to NH_3_ on the Pd/ACC catalyst. The average NH_3_ production rates and the corresponding FEs at various applied potentials are plotted in Figure 3(c) and [Supplementary-material supplementary-material-1]. The values obtained from the two methods were in good agreement ([Supplementary-material supplementary-material-1]). The average rate reached a maximum of 5.5 × 10^−11^ mol s^−1^ cm^−2^ at 0.1 V, which was normalized by the area of the working electrode. More importantly, a high FE of 97% for NH_3_ production was achieved, which may be attributed to the adjusted three-phase interface and the relative positive potential. The value was several times to one order of magnitude higher than that reported in aqueous solutions up to date ([Supplementary-material supplementary-material-1]). The NRR performances were evaluated with the same kind of Pd/ACC catalyst in N_2_-saturated HCl at constant voltage for 2 h for three times, and the results are shown in [Supplementary-material supplementary-material-1]. To demonstrate the almost inhibited HER, the produced H_2_ was then detected by gas chromatography and no signals of H_2_ was detected (note that the HER process can occur at positive potentials; see [Supplementary-material supplementary-material-1] and Supplemental Information: Calculation of equilibrium potential of HER). The metal nanoparticles were still homogenously dispersed on the ACC support after 2 h of continuous electrolysis ([Supplementary-material supplementary-material-1]). Recycling experiments also found that the chronoamperometry curves exhibited little change and the FE maintained around 90% during recycling for nine times, manifesting the repeatability and the stability of the adjusted three-phase interface ([Supplementary-material supplementary-material-1] and Figure 3(d)). The Pd/ACC catalyst after NRR was further characterized. Compared with pristine Pd/ACC and a reference sample prepared by heating Pd/ACC in a tube furnace with a N_2_ flow, the catalyst after NRR showed diffraction peaks slightly shifted toward lower diffraction angles in the XRD pattern and enlarged lattice distances in the HRTEM image, suggesting that Pd hydride might form after the NRR test (Figures [Supplementary-material supplementary-material-1]). The solid-state NMR measurement further confirmed it ([Supplementary-material supplementary-material-1]), consistent with the above DFT results and the previous reports that H atoms can enter into the lattice of Pd to form stable PdH under operating potentials [42, 43].

A series of control experiments were then performed to demonstrate that the detected NH_3_ was truly generated from the electrochemical NRR (Figures [Supplementary-material supplementary-material-1]). When Pd/ACC was evaluated in N_2_ at an open circuit, no signal of NH_3_ was detected, indicating the absence of NH_3_ impurity in the external environment and N_2_. Also, no NH_3_ was found after controlled electrolysis of Pd/ACC at 0.1 V in Ar for 2 h, which was consistent with the absence of N in the Pd/ACC XPS spectrum. In addition, no NH_3_ was produced on the pure ACC support. To further confirm the origin of NH_3_ production during the NRR test, an isotopic labeling experiment with ^15^N_2_ as the feed gas was conducted. As shown in Figure 3(e), the ^1^H nuclear magnetic resonance (^1^H NMR) spectrum of ^15^NH_4_^+^ shows a doublet coupling with a *J*_N‐H_ of 73.2 Hz, while a triple coupling with a *J*_N‐H_ of 52.2 Hz was found for ^14^NH_4_^+^. Only ^14^NH_4_^+^ was observed when ^14^N_2_ gas was fed into the cell. By contrast, the ^15^N_2_ sample showed distinguished peaks for ^15^NH_4_^+^, and no signal for ^14^NH_4_^+^ was observed in ^1^H NMR spectra, suggesting the negligible amount of background NH_3_ and coinciding with the control experiment under Ar electrolysis. These results all proved that the produced NH_3_ was entirely derived from the electrochemical reduction of N_2_.

### 2.4. Validity and Applicability of the Adjusted Three-Phase Interface

**Figure 4:**
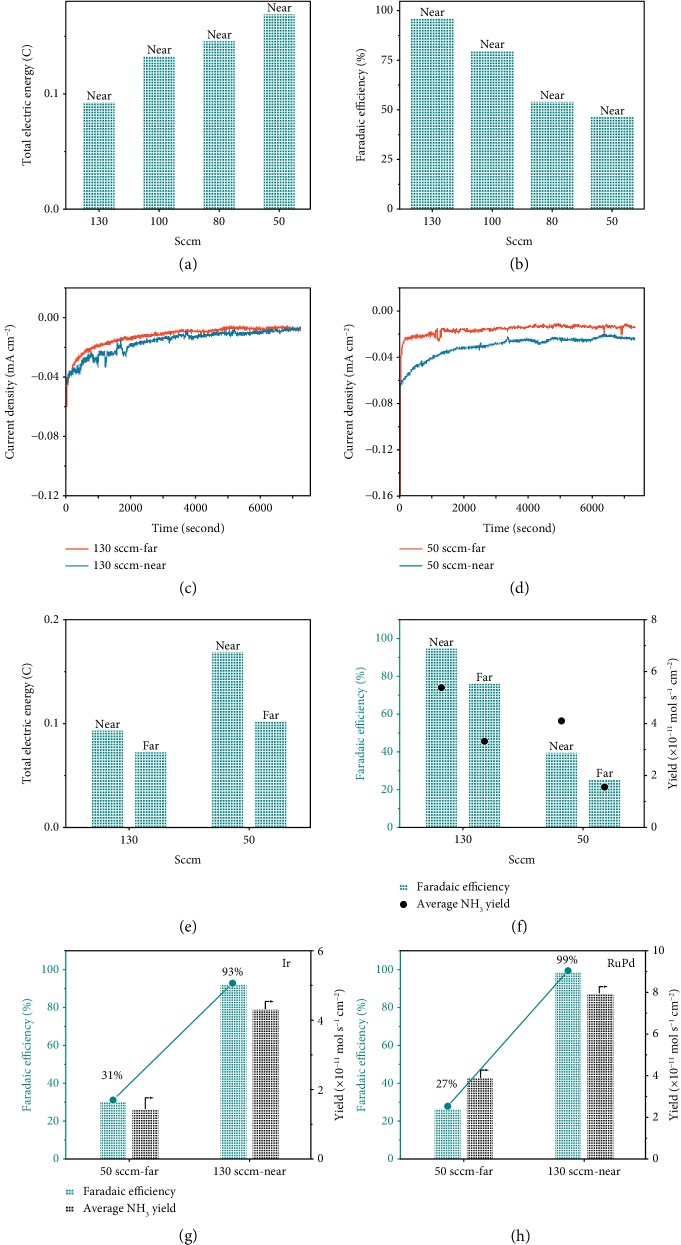
Effect of nitrogen bubbles on electrochemical NRR performances. (a) Total electric charge and (b) FE for Pd/ACC at 0.1 V under various flow rates of N_2_. Chronoamperometry curves of Pd/ACC at 0.1 V with a gas flow rate of (c) 130 and (d) 50 sccm. Gas tube far from the electrode (red curve) and gas tube near the electrode (blue curve). (e) Total electric charges and FEs and (f) average NH_3_ yield rates for Pd/ACC at 0.1 V for 2 h by changing the gas flow rate and the position of the gas tube. (g) FEs and average NH_3_ yield rates for (g) Ir/ACC and (h) RuPd/ACC at 0.1 V for 2 h by changing the gas flow rate and the position of the gas tube.

To prove the validity and the importance of the adjusted three-phase interface, we investigated the influence of N_2_ coverage on the NRR performance by altering the N_2_ flow rate (50, 80, 100, and 130 sccm) and controlling the position of the gas tube. The results show that the current densities and total electric charges diminish with the increase in the N_2_ flow rate and achieve the lowest values at 130 sccm (Figure 4(a) and [Supplementary-material supplementary-material-1]), while the FE exhibits an opposite trend (Figure 4(b)). To explore the side reaction along with the NRR process at 50 sccm of N_2_, the produced H_2_ was detected by gas chromatography. As shown in [Supplementary-material supplementary-material-1], the H_2_ signal is found, indicating that HER occurs at 50 sccm of N_2_. These phenomena all proved that the HER process was suppressed with the increase in bubbles accessible to the electrode under 130 sccm of N_2_. Moreover, if the gas tube was positioned near the Pd/ACC catalyst to ensure that N_2_ bubbles surrounded the electrode, the current densities increased quickly in the chronoamperometry curves (Figures 4(c)–4(e)). Also, FE and NH_3_ yield increased prominently (Figure 4(f)), manifesting that the NRR activity could be enhanced with the N_2_ bubbles covering. Based on the above results, the significantly improved NRR selectivity of the Pd/ACC catalyst can be attributed to the synergistic effect of inhibited HER and promoted NRR activity with the enhanced three-phase interface.

Furthermore, to demonstrate the feasibility of this strategy and explore the applicability of the adjusted three-phase interface on other catalysts, Ir-based and RuPd-based catalysts with strong H adsorption ability were prepared via an ultrasound-assisted reduction method and then characterized using XRD, XPS, and HRTEM techniques. The results are shown in Figures [Supplementary-material supplementary-material-1], and their corresponding NRR properties were then examined. Maximum FEs of 93% and 99% for NH_3_ synthesis were achieved with the Ir/ACC and RuPd/ACC catalysts, respectively (Figures 4(g) and 4(h)). Additionally, it was found that more N_2_ coverage on Ir and RuPd surfaces could accelerate NH_3_ production and inhibit the HER process simultaneously (Figures [Supplementary-material supplementary-material-1]), therefore improving the NRR selectivity and activity synergistically. These findings were consistent with those for Pd/ACC, which could open up a new outlook to break through the selectivity limitations in electrochemical NRR, especially in aqueous solution. In addition, it was reasonable to expect an excellent NRR performance with the enhanced three-phase interface by engineering catalysts with more active sites. We found that the developed Pd clusters/ACC and IrPd/ACC catalysts with supersmall metal nanoparticle sizes (Figures [Supplementary-material supplementary-material-1]) could achieve improved ammonia yield rates of 106 *μ*g h^−1^ mg_metal_^−1^ and 93 *μ*g h^−1^ mg_metal_^−1^, respectively, while the high FEs remains (97% and 87%, Figures [Supplementary-material supplementary-material-1]). These all demonstrated the wide applicability of the enhanced three-phase interface.

## 3. Discussion

In summary, we demonstrate a theory-guided design of the adjusted three-phase interface as an effective strategy for favoring NRR over HER and achieve high NH_3_ production selectivity with FE up to 97% in an aqueous acid solution under ambient condition. N_2_ does not have to be dissolved in electrolyte, and the increased N_2_ coverage on the catalyst surface weakens H adsorption, leading to a suppressed HER and improved selectivity toward NH_3_, as evidenced by the theoretical and experimental results. This strategy is also extended to Ir- and RuPd-based catalysts for selective reduction of N_2_ to NH_3_, even though they both possess strong H adsorption ability, thereby demonstrating its feasibility for electrochemical NRR especially in aqueous solutions. The NRR activity and selectivity can also be improved simultaneously by employing the three-phase interface and engineering catalysts with more active sites such as Pd clusters/ACC and IrPd/ACC. Thus, the results should provide a new perspective for enhancing the electrocatalytic performance of solid catalysts in a gas-involved and/or proton-sensitive competition system.

## 4. Materials and Methods

### 4.1. Chemicals

Sodium tetrachloropalladate (Cl_4_Na_2_Pd), ruthenium chloride hydrate (RuCl_3_·*x*H_2_O), chloroiridic acid hydrate (H_2_IrCl_6_·*x*H_2_O), sodium borohydride (NaBH_4_), hydrazine monohydrate (N_2_H_4_·H_2_O, >98% (T)), sodium nitroferricyanide dihydrate (C_5_FeN_6_Na_2_O·2H_2_O), sodium citrate (C_6_H_5_Na_3_O_7_), sodium salicylate (C_7_H_5_NaO_3_), sodium hypochlorite solution (NaClO, 5% Cl^−^), ammonium chloride (NH_4_Cl), and ^15^N enrichment of ammonium chloride (^15^NH_4_Cl, 99 atom% ^15^N) were purchased from Sigma-Aldrich. Hydrochloric acid (HCl, 35%-38%), sodium hydroxide (NaOH), and ethyl alcohol (C_2_H_5_OH) were purchased from Sinopharm Chemical Reagent Co. Ltd. Para-dimethylaminobenzaldehyde (C_9_H_11_NO) was purchased from Macklin Biochemical Co., Ltd. A nitrite assay kit was purchased from Nanjing Jiancheng Bioengineering Institute. Carbon cloth (HCCP330) was purchased from Shanghai Hesen Electric Co. Ltd. Nafion 211 membranes were purchased from Fuel Cell Store. Ultra-high-purity N_2_ gas (99.9999%) and ultra-high-purity Ar gas (99.999%) were purchased from Jingong Material Gas Co. Ltd. ^15^N_2_ gas (chemical purity: ≥98.5%) was purchased from Newradar Special Gas Co., Ltd. Ultrapure water with a resistivity of 18.2 MΩ cm was produced using a Millipore Milli-Q grade. All of the chemicals were used without any further purification.

### 4.2. Synthesis of ACC

ACC was synthesized according to the previous report [44]. Typically, a piece of CC was heated to 600°C with a heating rate of 10°C min^−1^ and kept at 600°C for 1 h under flowing industry N_2_ (99% purity) at 300 sccm to obtain ACC.

### 4.3. Synthesis of the Pd/ACC Catalyst

2.5 mL of 0.01 g mL^−1^ Cl_4_Na_2_Pd solution was dispersed in 20 mL of ultrapure water. A piece of ACC was immersed in the solution under ultrasound for 10 min. Next, 2.5 mL of 2 mg mL^−1^ NaBH_4_ solution was added in the as-prepared solution with an ultrasonic dispersion for 30 min. Finally, the as-obtained Pd/ACC catalyst was further washed and dried at 60°C in a vacuum oven. The content of Pd on the Pd/ACC catalyst was determined to be ~0.4 mg cm^−2^ according to the ICP-AES result.

### 4.4. Characterizations

The X-ray diffraction (XRD) data of the samples were obtained on D/tex-Ultima TV wide-angle X-ray diffractometer equipped with Cu K*α* radiation (*λ* = 0.15406 nm). High-resolution TEM (HRTEM) and high-angle annular dark-field scanning transmission electron microscopy (HADDF-STEM) analyses were performed on an FEI Tecnai G2 F20 S-TWIN microscope operating at an acceleration voltage of 300 kV. The X-ray photoelectron (XPS) data were accomplished with an ESCALAB MARK II spherical analyzer with an Al K*α* (Al 1486.6 eV) X-ray source. The inductively coupled plasma-atomic emission spectrometry (ICP-AES) was carried out on PerkinElmer Optima OES 800. The content of Pd was obtained with inductively coupled plasma-atomic emission spectrometry (ICP-AES, PerkinElmer Optima OES 8000), which was dissolved by HNO_3_. The concentration of ammonia was measured on a UV-vis spectrophotometer (TU-1901). The specific surface area and pore size distribution were calculated based on the N_2_ adsorption analysis performed at 77 K on Micromeritics ASAP 2020. 600 M ^1^H nuclear magnetic resonance (NMR) was detected on Agilent DD2-600. Diffuse reflectance infrared Fourier transform spectroscopy (DRIFTS) was collected with Nicolet 6700 FTIR fitted with an MCT detector with a resolution of 4 cm^−1^ and 32 scans. Gas chromatography was performed on Fuli 9790 equipped with tandem connect of PorparkQ and a thermal conductivity detector with Ar as the carrier gas (injector, oven, and detector temperatures were set at 150°C, 120°C, and 200°C, respectively).

### 4.5. Electrochemical NRR Measurements

The electrochemical experiments were carried out in a three-electrode configuration at an electrochemical station (CHI660E). Typically, the catalysts were freestanding, which were directly used as the working electrode without the addition of any additives. The area of the working electrode was controlled to be 0.8 cm^2^. Pt foil and Ag/AgCl electrode (saturated KCl electrolyte) were used as the counter electrode and reference electrode, respectively. In this work, all potentials were converted to reversible hydrogen electrode (RHE) scaling by *E* (vs. RHE) = E (vs. Ag/AgCl) + 0.1989 V + 0.0591 × pH.

For electrochemical NRR measurements, the tests were conducted in a two-compartment cell with 40 mL electrolyte in each cell which was separated with a Nafion 211 membrane and connected with a gas absorption cell. Before experiments, the Nafion 211 membrane was pretreated with H_2_O_2_ (5%) aqueous solution and ultrapure water each for 1 h. Ultra-high-purity N_2_ (99.9999% purity) was used as the feeding gas to reduce a false positive from potential contaminant from the input gas stream. In addition, before NRR tests, the potential NO*_x_* in ultra-high-purity N_2_ gas (99.9999% purity) was first detected with a nitrite assay kit by using colorimetric tests, gas chromatography, and DRIFTS (see Determination of NO*_x_* Contamination in Ultra-High-Purity N_2_ Gas). These results all show that almost no NO*_x_* in the feeding gas was detected. Then, the ultra-high-purity N_2_ was continuously purged into the cathodic compartment for at least 1 h before NRR tests. Then, potentiostatic tests were performed in N_2_-saturated 0.1 M HCl solution for 2 h at room temperature under atmosphere pressure for evaluating the average ammonia yield rate. The gas tube was positioned to ensure that the entire cathode was surrounded by gas bubbles during the whole process. In order to absorb the gas and electrolyte out with the large flow rate of N2, 0.1 M HCl was also used as an absorber in a gas absorption cell. To reduce the contaminations from the environment, the whole cell was sealed and soaked in 0.1 M HCl solution when not in use and it was thoroughly rinsed with deionized water and 0.1 M HCl electrolyte each for more than three times. Before LSV tests, Ar flowed into the cell for 1 h. Then, polarization curves in Ar were tested with a scan rate of 5 mV s^−1^, and this step was repeated to obtain a steady curve. Next, the gas was changed to ultra-high-purity N_2_ and it flowed in the cell to 1 h to guarantee a N_2_ atmosphere. For comparison, the LSV curves in N_2_ were obtained at 5 mV s^−1^ with the same cell assembly. Then, N_2_ gas was changed to Ar again and Ar was inputted in the cell for 1 h before collecting the LSV curves in N_2_ at 5 mV s^−1^. All the polarization curves shown were the steady ones. The chronoamperometry test of Ar was conducted on a Pd/ACC sample from the same batch of catalyst in 0.1 M Ar-saturated HCl at 0.1 V for 2 h with 130 sccm of Ar flow rate before the NRR test. Repeated tests were run on Pd/ACC with the same electrode or from different batches in 0.1 M HCl solution at constant voltage for 2 h with 130 sccm of N_2_ flow rate. Recycling experiments were repeated for nine times, and each was evaluated by NRR tests on the same Pd/ACC electrode in 0.1 M HCl solution at 0.1 V for 2 h with 130 sccm of N_2_ flow rate.

### 4.6. Determination of NO*_x_* Contamination in Ultra-High-Purity N_2_ Gas

The potential NO*_x_* in ultra-high-purity N_2_ gas should be noticed to avoid a false positive. The nitrite assay kit, gas chromatography, and DRIFTS were used to detect the potential NO*_x_* in the ultra-high-purity N_2_. Similar to the NRR test, N_2_ was purged into the electrolytic cell for 2 h, and then, the solution was detected with the nitrite assay kit according to the previous report [45]. Ultrapure water and 20 *μ*mol/L NaNO_2_ sample added with nitrite assay kits were also prepared as the blank sample and standard sample, respectively. After color development for 15 min at room temperature, these samples were measured at a wavelength of 546 nm on a UV-vis spectrophotometer. As shown in [Supplementary-material supplementary-material-1], the UV-vis spectrum of the test solution is consistent with that of the blank solution. The concentration of nitrite in the test solution was low to 1 ppb, and thus, the NO*_x_* in the ultra-high-purity N_2_ could be ignored. Also, the feeding gas was further detected by gas chromatography and DRIFTS. Ar was used as the carrier gas, and the flow rate was 30 sccm for gas chromatographic analysis. As shown in [Supplementary-material supplementary-material-1], only the peak of N_2_ is found and no other peak is detected, indicating a negligible NO*_x_* in the N_2_ feeding gas. For DRIFTS, Ar was used as the background and the final spectrum of N_2_ was obtained by subtracting that from the Ar background ([Supplementary-material supplementary-material-1]). Similarly, no vibrations of NO, NO_2_, and other NO*_x_* were observed. The results all demonstrated the negligible NO*_x_* in the feeding N_2_.

### 4.7. Determination of Ammonia

#### 4.7.1. Indophenol Blue Method

Concentration of ammonia was determined by the indophenol blue method. In detail, 3 mL of solution after NRR tests was first removed to a colorimetric tube. Then, 3 mL of 1 M NaOH solution containing 5 wt% sodium citrate and 5.79 wt% sodium salicylate was added, followed by the addition of 1.5 mL of 0.05 M NaClO and 0.3 mL of 1 wt% sodium nitroferricyanide dehydrate. After color development for 2 h at room temperature, the adsorption spectrum was obtained on a UV-vis spectrophotometer. The absorbance at a wavelength of 655 nm was used for qualitative determination of indophenol blue. The blank control was prepared by replacing 3 mL of sample with 3 mL of 0.1 M HCl solution without ammonia. The concentration-absorbance curve was calibrated using standard ammonia chloride solutions with a series of concentrations. To reduce other influence factors, the background was corrected by subtracting the value of the blank control from all readings of samples.

#### 4.7.2. Salicylic Acid Method

Concentration of ammonia was also confirmed with the salicylic acid method [8]. 4 mL of solution after NRR tests was first removed to an empty bottle. Then, 4 mL of ultrapure water was added, followed by the addition of 50 *μ*L of oxidizing solution containing 0.75 M NaOH and NaClO (*ρ*_Cl_ = 4‐4.9) and 500 *μ*L of coloring solution containing 0.4 M sodium salicylate and 0.32 M NaOH. Finally, 50 *μ*L of catalyst solution containing 1 wt% sodium nitroferricyanide dehydrate was added. After color development for 1.5 h at room temperature, the adsorption measurement was conducted at a wavelength of 670 nm on a UV-vis spectrophotometer. The blank control was prepared by replacing 4 mL of sample with 4 mL of 0.1 M HCl solution without ammonia. The concentration-absorbance curve was calibrated using standard ammonia chloride solutions with a series of concentrations. To reduce other influence factors, the background was corrected by subtracting the value of the blank control from all readings of samples.

### 4.8. Determination of Hydrazine

#### 4.8.1. Para-dimethylaminobenzaldehyde Method

The hydrazine present was estimated by the method of Watt and Chrisp [46]. A mixture of para-dimethylaminobenzaldehyde (5.99 g) and HCl (concentrated, 30 mL) and ethanol (300 mL) was used as a color reagent. First, 2 mL of solution after NRR tests was mixed with 2 mL water. Then, 1 mL of 1 M KOH solution and 5 mL of color reagent were added, followed by sitting for 10 min at room temperature for color development. The absorbance of the resulting solution was measured at 456 nm. The blank control was prepared by replacing 2 mL of sample with 2 mL of 0.1 M HCl solution without hydrazine. The concentration-absorbance curve was calibrated using standard hydrazine hydrate solutions with a series of concentrations. To reduce other influence factors, the background was corrected by subtracting the value of the blank control from all readings of samples. The calibration curve was plotted with 2 mL of hydrazine hydrate-water solution of certain concentration, 2 mL of 0.1 M HCl solution, 1 mL of 1 M KOH solution, and 5 mL of color reagent.

#### 4.8.2. ^15^N_2_ Isotope Labeling Experiments

^15^N_2_ was used as the feeding gas for the isotopic labeling experiment to confirm the source of ammonia. ^15^N_2_ (≥98.5% chemical purity) was purchased from Newradar Special Gas Co., Ltd. Before the NRR test, ^15^N_2_ was fed into the electrolytic cell with a flow rate of 10 sccm for 30 min. Pd/ACC was tested at 0.1 V vs. RHE for 4 h in the airtight device. This procedure was repeated for 2 times. The obtained acid electrolyte solution was concentrated, added with D_2_O, and then identified by 600 M ^1^H NMR (Agilent, DD2-600).

#### 4.8.3. DFT Calculations

DFT calculations were carried out using the Vienna Ab initio Simulation Package (VASP) [47] according to the projector augmented wave (PAW) method [48]. A cutoff energy of 400 eV for plane waves was set through all the calculations, and exchange-correlation functional approximation was treated in Perdew-Burke-Ernzerhof (PBE) functional [49]. The (211) slabs were built with 4 atomic layers in a *p*(1 × 3) supercell with the bottom two layers fixed during structural relaxation. A *p*(3 × 3) supercell containing 4 atomic layers for (111) and (100) slabs was modeled with the bottom two layers fixed during structural relaxation. The periodic condition was employed along the *x* and *y* directions, and the vacuum space along the *z* direction was set to be 15 Å in all slab calculations. The Monkhorst-Pack scheme was used for sampling the Brillouin zone, and the *k*-point grid of 3 × 3 × 1 is selected. During structural optimizations, the residual force between atoms was converged to a value below 0.02 eV/Å.

The average adsorption energies for N_2_ chemisorption with given N_2_ coverage are defined as

(1) $$\begin{array}{c} E_{\text{ads}}\left({\text{N}}_{2}\right)=\frac{E_{\text{tot}}1-E_{\text{slab}}-E_{{\text{N}}_{2}}}{n\left({\text{N}}_{2}\right)}, \end{array}$$

where *n*(N_2_) is the number of N_2_ adsorbed on the catalyst, *E*_tot_1 is the total energy after *n*(N_2_) of N_2_ is adsorbed on the catalyst, *E*_slab_ is the energy of the clean catalyst alone, and *E*_N_2__ is the energy of the molecule N_2_ in the gas phase.

The adsorption energies for H atom chemisorption are defined as

(2) $$\begin{array}{c} E_{\text{ads}}\left(\text{H}\right)=E_{\text{tot}}2-E_{\text{tot}}1-E_{\text{H}}, \end{array}$$

where *E*_tot_2 is the total energy after an H atom is adsorbed on the catalyst with given N_2_ coverage and *E*_H_ is the half of the energy of the molecule H_2_ in the gas phase.

## Supplementary Materials

Supplementary Materials Supplemental materials and methods. Figure S1: DFT calculations of *E*_ads_(N_2_) on Pd(111), Pd(100), and Pd(211) facets with vertical and parallel adsorption configuration of N_2_. Figure S2: DFT calculations on the effect of *θ*_N_2__ on *E*_ads_(H) on the Pd(211) surface. Figure S3: DFT calculations on the effect of *θ*_N_2__ on *E*_ads_(H) on the Pd(111) surface. Figure S4: N_2_ adsorption and desorption analysis of ACC. Figure S5: XPS spectrum of Pd/ACC. Figure S6: UV-vis adsorption spectrum of nitrite detection. Figure S7: gas chromatographic curve of ultra-high-purity N_2_. Figure S8: DRIFT spectrum of ultra-high-purity N_2_ by subtracting the signal of the Ar background. Figure S9: LSV curves of Pd/ACC under Ar and N_2_ condition. Figure S10: calibration of the indophenol blue method using standard ammonia chloride solutions with a series of concentrations. Figure S11: calibration curve of the salicylic acid method using standard ammonia chloride solutions with a series of concentrations. Figure S12: calibration curve for N_2_H_4_ using standard N_2_H_4_ solutions with a series of concentrations. Figure S13: UV-vis adsorption spectra of N_2_H_4_ detection in HCl solution after 2 h electrolysis. Figure S14: chronoamperometry curves and average yield rate of NH_3_ production of Pd/ACC at various potentials for 2 h. Figure S15: Faradic efficiency and yield rate of NH_3_ production on Pd/ACC based on the salicylic acid method. Figure S16: repeated tests of Pd/ACC for three times. Figure S17: gas chromatographic curves (Fuli 9790) for H_2_ detection during the NRR test at 0.1 V under 130 and 50 sccm of N_2_. Figure S18: HRTEM images of Pd/ACC after N_2_ electrochemical reduction. Figure S19: chronoamperometry results of Pd/ACC in N_2_-saturated HCl at 0.1 V vs. RHE for cycles. Figure S20: XRD spectra of Pd/ACC before and after the NRR test at 0.1 V. Figure S21: HRTEM image of Pd/ACC after the NRR test at 0.1 V. Figure S22: XRD image of Pd/ACC after heating in N_2_ in a tube furnace, compared with that of the pristine Pd/ACC. Figure S23: HRTEM image of Pd/ACC after sweeping in an N_2_ atmosphere in a tube furnace. Figure S24: solid-state ^1^H NMR spectra of Pd/ACC before and after the NRR test at 0.1 V. Figure S25: chronoamperometry results of ACC in N_2_-saturated HCl and Pd/ACC in Ar-saturated HCl at 0.1 V. Figure S26: UV-vis adsorption spectra of NH_4+_ detection in a gas absorber after 2 h electrolysis of contrast samples. Figure S27: LSV curves in N_2_-saturated HCl at a gas flow rate of 50 sccm and 130 sccm with bubbles covering the catalyst. Figure S28: FEs for Pd/ACC at 0.1 V under various flow rates of N_2_. Figure S29: XRD patterns of Ir/ACC and RuPd/ACC. Figure S30: XPS spectra. Figure S31: HRTEM images. Figure S32: chronoamperometry results of Ir/ACC. Figure S33: UV-vis adsorption spectra of NH_4+_ detection of the Ir/ACC catalyst. Figure S34: chronoamperometry results of RuPd/ACC. Figure S35: UV-vis adsorption spectra of NH_4+_ detection of the RuPd/ACC catalyst. Figure S36: XRD patterns of IrPd/ACC and Pd clusters/ACC. Figure S37: HAADF-STEM image of IrPd/ACC. Figure S38: HAADF-STEM image of Pd clusters/ACC. Figure S39: chronoamperometry result of IrPd/ACC. Figure S40: chronoamperometry result of Pd clusters/ACC. Figure S41: FEs and average NH_3_ yields of Pd clusters/ACC and IrPd/ACC at 0.1 V with the adjusted three-phase interface. Table S1: comparison of the NRR performance of Pd/ACC with other catalysts recently reported under ambient conditions. Movie S1: electrochemical NRR test with the adjusted three-phase interface.

## References

[1] Chen J. G., Crooks R. M., Seefeldt L. C. (2018). Beyond fossil fuel-driven nitrogen transformations. *Science*.

[2] van der Ham C. J. M., Koper M. T. M., Hetterscheid D. G. H. (2014). Challenges in reduction of dinitrogen by proton and electron transfer. *Chemical Society Reviews*.

[3] Guo C., Ran J., Vasileff A., Qiao S. Z. (2018). Rational design of electrocatalysts and photo(electro) catalysts for nitrogen reduction to ammonia (NH_3_) under ambient conditions. *Energy & Environmental Science*.

[4] Kyriakou V., Garagounis I., Vasileiou E., Vourros A., Stoukides M. (2017). Progress in the electrochemical synthesis of ammonia. *Catalysis Today*.

[5] Martin A. J., Shinagawa T., Perez-Ramirez J. (2019). Electrocatalytic reduction of nitrogen: from Haber-Bosch to ammonia artificial leaf. *Chem*.

[6] Légaré M.-A., Bélanger-Chabot G., Dewhurst R. D. (2018). Nitrogen fixation and reduction at boron. *Science*.

[7] Kordali V., Kyriacou G., Lambrou C. (2000). Electrochemical synthesis of ammonia at atmospheric pressure and low temperature in a solid polymer electrolyte cell. *Chemical Communications*.

[8] Chen S., Perathoner S., Ampelli C., Mebrahtu C., Su D., Centi G. (2017). Electrocatalytic synthesis of ammonia at room temperature and atmospheric pressure from water and nitrogen on a carbon-nanotube-based electrocatalyst. *Angewandte Chemie, International Edition*.

[9] Montoya J. H., Tsai C., Vojvodic A., Nørskov J. K. (2015). The challenge of electrochemical ammonia synthesis: a new perspective on the role of nitrogen scaling relations. *ChemSusChem*.

[10] Skúlason E., Bligaard T., Gudmundsdóttir S. (2012). A theoretical evaluation of possible transition metal electro-catalysts for N_2_ reduction. *Physical Chemistry Chemical Physics*.

[11] Chen G.-F., Cao X., Wu S. (2017). Ammonia electrosynthesis with high selectivity under ambient conditions via a Li^+^ incorporation strategy. *Journal of the American Chemical Society*.

[12] Kumar D., Pal S., Krishnamurty S. (2016). N_2_ activation on Al metal clusters: catalyzing role of BN-doped graphene support. *Physical Chemistry Chemical Physics*.

[13] Bezdek M. J., Chirik P. J. (2018). Interconversion of molybdenum imido and amido complexes by proton-coupled electron transfer. *Angewandte Chemie, International Edition*.

[14] Tang C., Qiao S.-Z. (2019). How to explore ambient electrocatalytic nitrogen reduction reliably and insightfully. *Chemical Society Reviews*.

[15] Shi M.-M., Bao D., Wulan B.-R. (2017). Au sub-nanoclusters on TiO_2_ toward highly efficient and selective electrocatalyst for N_2_ conversion to NH_3_ at ambient conditions. *Advanced Materials*.

[16] Zhang L., Ding L.-X., Chen G.-F., Yang X., Wang H. (2019). Ammonia synthesis under ambient conditions: selective electroreduction of dinitrogen to ammonia on black phosphorus nanosheets. *Angewandte Chemie, International Edition*.

[17] Mao J., Chen W., Sun W. (2017). Rational control of the selectivity of a ruthenium catalyst for hydrogenation of 4-nitrostyrene by strain regulation. *Angewandte Chemie, International Edition*.

[18] Tao H., Choi C., Ding L. X. (2019). Nitrogen fixation by Ru single-atom electrocatalytic reduction. *Chem*.

[19] Foster S. L., Bakovic S. I. P., Duda R. D. (2018). Catalysts for nitrogen reduction to ammonia. *Nature Catalysis*.

[20] Yu X., Han P., Wei Z. (2018). Boron-doped graphene for electrocatalytic N_2_ reduction. *Joule*.

[21] Wang J., Yu L., Hu L., Chen G., Xin H., Feng X. (2018). Ambient ammonia synthesis via palladium-catalyzed electrohydrogenation of dinitrogen at low overpotential. *Nature Communications*.

[22] Yang X., Nash J., Anibal J. (2018). Mechanistic insights into electrochemical nitrogen reduction reaction on vanadium nitride nanoparticles. *Journal of the American Chemical Society*.

[23] Geng Z., Liu Y., Kong X. (2018). Achieving a record-high yield rate of 120.9^*μg*_NH_3__ *mg*_cat._^−1^ h^−1^^ for N_2_ electrochemical reduction over Ru single-atom catalysts. *Advanced Materials*.

[24] Qiu W., Xie X. Y., Qiu J. (2018). High-performance artificial nitrogen fixation at ambient conditions using a metal-free electrocatalyst. *Nature Communications*.

[25] Zhang Y., Qiu W., Ma Y. (2018). High-performance electrohydrogenation of N_2_ to NH_3_ catalyzed by multishelled hollow Cr_2_O_3_ microspheres under ambient conditions. *ACS Catalysis*.

[26] Li S.-J., Bao D., Shi M. M., Wulan B. R., Yan J. M., Jiang Q. (2017). Amorphizing of Au nanoparticles by CeO*_X_*-RGO hybrid support towards highly efficient electrocatalyst for N_2_ reduction under ambient conditions. *Advanced Materials*.

[27] Luo Y., Chen G. F., Ding L., Chen X., Ding L. X., Wang H. (2019). Efficient electrocatalytic N_2_ fixation with MXene under ambient conditions. *Joule*.

[28] Chen J., Wang H., Wang Z. (2019). Redispersion of Mo-based catalysts and the rational design of super small-sized metallic Mo species. *ACS Catalysis*.

[29] Choi C., Back S., Kim N. Y., Lim J., Kim Y. H., Jung Y. (2018). Suppression of hydrogen evolution reaction in electrochemical N_2_ reduction using single-atom catalysts: a computational guideline. *ACS Catalysis*.

[30] Wang M., Liu S., Qian T. (2019). Over 56.55% Faradaic efficiency of ambient ammonia synthesis enabled by positively shifting the reaction potential. *Nature Communications*.

[31] Singh A. R., Rohr B. A., Schwalbe J. A. (2017). Electrochemical ammonia synthesis-the selectivity challenge. *ACS Catalysis*.

[32] Tanaskovic V., Pasti I., Mentus S. (2013). Polycrystalline platinum rotating disc electrode study of the liquid system 0.2 M LiClO_4_-H_2_O-DMSO in nitrogen and oxygen atmosphere. *International Journal of Electrochemical Science*.

[33] Cheng H., Ding L. X., Chen G. F., Zhang L., Xue J., Wang H. (2018). Molybdenum carbide nanodots enable efficient electrocatalytic nitrogen fixation under ambient conditions. *Advanced Materials*.

[34] Lee H. K., Koh C. S. L., Lee Y. H. (2018). Favoring the unfavored: selective electrochemical nitrogen fixation using a reticular chemistry approach. *Science Advances*.

[35] Yan L., Li D., Yan T. (2018). N,P,S-codoped hierarchically porous carbon spheres with well-balanced gravimetric/volumetric capacitance for supercapacitors. *ACS Sustainable Chemistry & Engineering*.

[36] Li J., Zhu Y., Chen W. (2019). Breathing-mimicking electrocatalysis for oxygen evolution and reduction. *Joule*.

[37] Zhou F., Azofra L. M., Ali M. (2017). Electro-synthesis of ammonia from nitrogen at ambient temperature and pressure in ionic liquids. *Energy & Environmental Science*.

[38] Li J., Chen G., Zhu Y. (2018). Efficient electrocatalytic CO_2_ reduction on a three-phase interface. *Nature Catalysis*.

[39] Singh M. R., Kwon Y., Lum Y., Ager J. W., Bell A. T. (2016). Hydrolysis of electrolyte cations enhances the electrochemical reduction of CO_2_ over Ag and Cu. *Journal of the American Chemical Society*.

[40] Amar I. A., Lan R., Petit C. T. G., Tao S. (2011). Solid-state electrochemical synthesis of ammonia: a review. *Journal of Solid State Electrochemistry*.

[41] Yao Y., Wang H., Yuan X.-Z., Li H., Shao M. (2019). Electrochemical nitrogen reduction reaction on ruthenium. *ACS Energy Letters*.

[42] Wickman B., Fredriksson M., Feng L., Lindahl N., Hagberg J., Langhammer C. (2015). Depth probing of the hydride formation process in thin Pd films by combined electrochemistry and fiber optics-based *in situ* UV/vis spectroscopy. *Physical Chemistry Chemical Physics*.

[43] Sheng W., Kattel S., Yao S. (2017). Electrochemical reduction of CO_2_ to synthesis gas with controlled CO/H_2_ ratios. *Energy & Environmental Science*.

[44] Wang H., Deng J., Xu C. (2017). Ultramicroporous carbon cloth for flexible energy storage with high areal capacitance. *Energy Storage Materials*.

[45] Andersen S. Z., Čolić V., Yang S. (2019). A rigorous electrochemical ammonia synthesis protocol with quantitative isotope measurements. *Nature*.

[46] Watt G. W., Chrisp J. D. (1952). A spectrophotometric method for the determination of hydrazine. *Analytical Chemistry*.

[47] Kresse G., Furthmüller J. (1996). Efficiency of ab-initio total energy calculations for metals and semiconductors using a plane-wave basis set. *Computational Materials Science*.

[48] Blochl P. E. (1994). Projector augmented-wave method. *Physical Review B*.

[49] Hammer B., Hansen L. B., Norskov J. K. (1999). Improved adsorption energetics within density-functional theory using revised Perdew-Burke-Ernzerhof functionals. *Physical Review B*.

