# Supplementary material for: Selective Electrochemical Reduction of Nitrogen to Ammonia by Adjusting the Three-Phase Interface
Source: Research (Wash D C). 2019 Nov 30;2019:1401209. doi: 10.34133/2019/1401209 (PMC6944493; doi:10.34133/2019/1401209)
Supplement: Supplementary Materials — Supplemental materials and methods. Figure S1: DFT calculations of Eads(N2) on Pd(111), Pd(100), and Pd(211) facets with vertical and parallel adsorption configuration of N2. Figure S2: DFT calculations on the effect of θN2 on Eads(H) on the Pd(211) surface. Figure S3: DFT calculations on the effect of θN2 on Eads(H) on the Pd(111) surface. Figure S4: N2 adsorption and desorption analysis of ACC. Figure S5: XPS spectrum of Pd/ACC. Figure S6: UV-vis adsorption spectrum of nitrite detection. Figure S7: gas chromatographic curve of ultra-high-purity N2. Figure S8: DRIFT spectrum of ultra-high-purity N2 by subtracting the signal of the Ar background. Figure S9: LSV curves of Pd/ACC under Ar and N2 condition. Figure S10: calibration of the indophenol blue method using standard ammonia chloride solutions with a series of concentrations. Figure S11: calibration curve of the salicylic acid method using standard ammonia chloride solutions with a series of concentrations. Figure S12: calibration curve for N2H4 using standard N2H4 solutions with a series of concentrations. Figure S13: UV-vis adsorption spectra of N2H4 detection in HCl solution after 2 h electrolysis. Figure S14: chronoamperometry curves and average yield rate of NH3 production of Pd/ACC at various potentials for 2 h. Figure S15: Faradic efficiency and yield rate of NH3 production on Pd/ACC based on the salicylic acid method. Figure S16: repeated tests of Pd/ACC for three times. Figure S17: gas chromatographic curves (Fuli 9790) for H2 detection during the NRR test at 0.1 V under 130 and 50 sccm of N2. Figure S18: HRTEM images of Pd/ACC after N2 electrochemical reduction. Figure S19: chronoamperometry results of Pd/ACC in N2-saturated HCl at 0.1 V vs. RHE for cycles. Figure S20: XRD spectra of Pd/ACC before and after the NRR test at 0.1 V. Figure S21: HRTEM image of Pd/ACC after the NRR test at 0.1 V. Figure S22: XRD image of Pd/ACC after heating in N2 in a tube furnace, compared with that of the pristine Pd/AC [file 1401209.f1.zip › supplemental information for publication.docx]

Supplemental Information

Selective Electrochemical Reduction of Nitrogen to Ammonia by Adjusting the Three-Phase Interface

Haiyan Wang, Yuzhuo Chen, Ruxue Fan, Jiadong Chen, Zhe Wang, Shanjun Mao, Yong Wang*

**Supplemental Materials and Methods**

**Synthesis of Ir/ACC catalyst:** The synthesis procedure of Ir/ACC catalyst is the same to that of Pd/ACC by replacing Cl_4_Na_2_Pd solution with H_2_IrCl_6_·xH_2_O.

**Synthesis of RuPd/ACC catalyst:** 2.5 mL of 0.01 g mL^-1^ RuCl_3_·xH_2_O solution was dispersed in 20 mL of ultrapure water. A piece of as-prepared ACC was immersed in the solution under [ultrasound](javascript:void(0);) for 10 min. Next, 2.5 mL of 2 mg mL^-1^ NaBH_4_ solution was added in the as-prepared solution with an ultrasonic dispersion for 30 min. The as-obtained Ru/ACC was further washed with ultrapure water for several times. Then 2.5 mL of 0.01 g mL^-1^ Cl_4_Na_2_Pd solution was dispersed in 20 mL of ultrapure water to obtain Pd solution. Then Ru/ACC catalyst was immersed in the solution under [ultrasound](javascript:void(0);) for 10 min. Next, 2.5 mL of 2 mg mL^-1^ NaBH_4_ solution was added in the as-prepared solution with an ultrasonic dispersion for 30 min to obtain RuPd/ACC catalyst. Finally, the catalyst was washed with ultrapure water for several times and dried at 60 ^o^C in a vacuum oven.

**Synthesis of IrPd/ACC catalyst:** 300 uL of 0.01 g mL^-1^ H_2_IrCl_6_·xH_2_O solution was dispersed in 40 mL of ultrapure water. A piece of ACC was immersed in the solution under [ultrasound](javascript:void(0);) for 10 min. Next, 1 mL of 2 mg mL^-1^ NaBH_4_ solution was added in the as-prepared solution with an ultrasonic dispersion for 30 min. The as-obtained Ir/ACC was further washed with ultrapure water for several times. Then 300 uL of 0.01 g mL^-1^ Cl_4_Na_2_Pd solution was dispersed in 40 mL of ultrapure water and the Ir/ACC catalyst was immersed in the solution under [ultrasound](javascript:void(0);) for 10 min. Next, 1 mL of 2 mg mL^-1^ NaBH_4_ solution was added in the as-prepared solution with an ultrasonic dispersion for 30 min to obtain IrPd/ACC catalyst. Finally, the catalyst was washed with ultrapure water for several times and dried at 60 ^o^C in a vacuum oven. The whole content of Ir and Pd on IrPd/ACC catalyst was determined to be 60 ug cm^-2^ according to the ICP-AES result.

**Synthesis of Pd clusterts /ACC catalyst:** 300 uL of 0.01 g mL^-1^ Cl_4_Na_2_Pd solution was dispersed in 40 mL of ultrapure water. A piece of ACC was immersed in the solution under [ultrasound](javascript:void(0);) for 10 min and then taken out. Next, the as-prepared material was washed with ultrapure water for several times. The as-obtained material was further immersed in 40 mL of ultrapure water and then 1 mL of 2 mg mL^-1^ NaBH_4_ solution was added with an ultrasonic dispersion for 30 min. Finally, the catalyst was washed with ultrapure water for several times and dried at 60 ^o^C in a vacuum oven. The content of Pd on Pd clusters/ACC was determined to be 26 ug cm^-2^ according to the ICP-AES result.

**Statistic results**

The chrono-amperometry tests were run on Pd/ACC from different batches in N_2_-saturated HCl at constant voltage for 2 h with a N_2_ flow rate of 130 sccm for three times. The recycling NRR experiments were conducted on a Pd/ACC electrode tested at 0.1 V for 2 h with a N_2_ flow rate of 130 sccm for nine times. The electrochemcial NRR tests of different catalysts such as Ir/ACC, RuPd/ACC, Pd clusters/ACC and IrPd/ACC catalysts were based on the chrono-amperometry tests in N_2_-saturated HCl at 0.1 V for 2 h with a N_2_ flow rate of 130 sccm for more than three times.

**Calculation of equilibrium potential of NRR**

The standard potential for the half reaction of N_2_ reduction to NH_4_OH was 0.058 V vs.RHE at 298.15 K. The equilibrium potential is calculated using the Nernst equation, assuming 1 atm of N_2_ and 10^-6^ M of NH_4_OH in the solution.

$$\text{N}_{\text{2}}\left( \text{g} \right)\text{+2}\text{H}_{\text{2}}\text{O+6}\text{H}^{\text{+}}\text{+6}\text{e}^{\text{-}}\text{→2}\text{NH}_{\text{4}}\text{OH }\left( \text{aq} \right)\text{ }\text{E}^{\text{o}}\text{=0.058 V}\text{ }\text{vs. }\text{RHE}$$

Where F is the Faraday constant.

The equilibrium potential E:

$$\text{E=E}^{\text{o}}\text{-}\frac{\text{RT}}{\text{6F}}\ln\left( \frac{\left[ \text{NH}_{\text{4}}\text{OH} \right]^{\text{2}}}{\left[ \text{H}^{\text{+}} \right]^{\text{6}}} \right)\text{+}\text{0.059×pH}\text{=0.1}\text{7}\text{6 V vs. }\text{RHE}$$

**Calculation of equilibrium potential of HER**

$$\text{H}^{\text{+}}\left( \text{aq} \right)\text{ +}{\text{2}\text{e}}^{\text{-}}\text{→}H_{2}\text{ }\left( \text{g} \right)\text{ }\text{E}^{\text{0}}\text{=}\text{ }\text{0}\text{ }\text{V}$$

The equilibrium potential E(H^+^/H_2_):

$${\text{E}\text{(H}\text{+}\text{/H}\text{2}\text{)}\text{=E}}^{0}\text{-}\frac{\text{RT}}{\text{2}\text{F}}\ln\left( \frac{{(p}_{H_{2}}/p^{\theta})}{\left[ \text{H}^{\text{+}} \right]} \right)\text{+}\text{0.059×pH}\text{=}\text{-}\frac{\text{RT}}{2F}\text{ln }{(p}_{H_{2}}/p^{\theta}\text{) }\text{V }\text{ }\text{vs. }\text{RHE}$$

Where F is the Faraday constant, *p*(H_2_) is the hydrogen pressure .

Therefore, the equilibrium potential of HER is associated with *p*(H_2_). In the present case, the hydrogen is much lower than the standard atmospheric pressure *p^θ^* (1 bar) and the HER happens at positive potentials [[1](#_ENREF_1)].

Assuming *p*(H_2_)=2×10^-5^ bar at 0.075 V vs. RHE based on the experiment, the equilibrium potential is calculated using the Nernst equation. The equilibrium potential is shifted to 0.13V vs. RHE.

**Determination of ammonia yield rate and Faradaic efficiency**

**Calculation of Ammonia yield rate:** The ammonia yield rate was calculated based on spectrophotometry results of the cathodic cell and gas absorption cell with indophenol blue method. The rate of NH_3_ formation can be calculated as follows:

$$\text{r}_{\text{NH}_{\text{3}}}\text{(}\text{mol}\text{ }\text{s}^{\text{-1}}\text{ }\text{cm}^{\text{-2}}\text{)=}\frac{\text{C}_{{\text{electrolyte,}\text{ }\text{NH}}_{\text{3}}}\text{×}\text{V}_{\text{1}}\text{+}\text{C}_{{\text{absorber,}\text{ }\text{NH}}_{\text{3}}}\text{×}\text{V}_{\text{2}}}{\text{17t×A}}$$

where C_electrolyte,NH3_ is the measured NH_3_ concentration in the cathodic cell (g mL^-1^), *V_1_* is the volume of the HCl solution in the cathodic cell (mL), C_adsorber,NH3_is the measured NH_3_ concentration in the gas absorption cell (g mL^-1^), *V_2_* is the volume of the HCl solution in the gas absorption cell(mL), t is the time of collection (s) and A is the geometric area of the active electrode (cm^-2^).

$$\text{r}_{\text{NH}_{\text{3}}}\text{(}\text{ug}\text{ }\text{h}^{\text{-1}}\text{ }\text{mg}^{\text{-1}}\text{)=}\frac{\text{C}_{\text{electrolyte,NH}_{\text{3}}}\text{×}\text{V}_{\text{1}}\text{+}\text{C}_{\text{absorber,NH}_{\text{3}}}\text{×}\text{V}_{\text{2}}}{\text{t×m}}$$

where C_electrolyte,NH3_ is the measured NH_3_ concentration in the cathodic cell (ug mL^-1^), *V_1_* is the volume of the HCl solution in the cathodic cell (mL), C_adsorber,NH3_ is the measured NH_3_ concentration in the gas absorption cell (ug mL^-1^), *V_2_* is the volume of the HCl solution in the gas absorption cell(mL), t is the time of collection (h) and m is the mass of Pd on the active electrode(mg).

**Faradaic efficiency (FE):** The FE for NRR was defined as the electric quantity of NH_3_ synthesis divided the quantity of electric charge passed through the electrode during the electrolysis. The total amount of NH_3_-N produced in the cathodic cell and gas absorption cell was calculated based on the indophenol blue spectrophotometry methods. The faradaic efficiency for synthesis of NH_3_ can be calculated as follows:

$$\text{FE=}\frac{\text{3(C}_{{\text{electrolyte,}\text{ }\text{NH}}_{\text{3}}}\text{×}\text{V}_{\text{1}}\text{+}\text{C}_{{\text{absorber,}\text{ }\text{NH}}_{\text{3}}}\text{×}\text{V}_{\text{2}}\text{)×F}}{\text{17Q}}$$

where C_electrolyte,NH3_ is the measured NH_3_ concentration in the cathodic cell (g mL^-1^), *V_1_* is the volume of the HCl solution in the cathodic cell (mL), C_adsorber,NH3_ is the measured NH_3_ concentration in the gas absorption cell (g mL^-1^), *V_2_* is the volume of the HCl solution in the gas absorption cell (mL), F is Faradaic constant, 96485 C mol^-1^ and Q is the total electric charge integrated by i-t curve (C).

**Effect of *θ_N2_* on selectivity**

Based on the simple model of electrochemical N_2_ reduction process proposed by previous report [[2](#_ENREF_2)]:

$\text{H}^{\text{+}}\text{+}\text{e}^{\text{-}}\text{+*}\text{N}_{\text{2}}\underset{\leftrightarrow}{\text{k}_{\text{N}}}\text{*}\text{N}_{\text{2}}\text{H}$ (R1)

$\text{H}^{\text{+}}\text{+}\text{e}^{\text{-}}\text{+*H}\underset{\leftrightarrow}{\text{k}_{\text{H}}}\text{H}_{\text{2}}\text{+*}$ (R2)

The rate of ammonia and hydrogen production can be written as:

$\text{r}_{\text{N}}\text{=}\text{k}_{\text{N}}{\text{θ}_{\text{N}}\text{c'}}_{\text{+}}\text{c'}_{\text{-}}$ (1)

$r_{H}=k_{H}\theta_{H}{c'}_{+}{c'}_{-}$ (2)

where *k_N_* and *k_H_* are the rate constants of R_1_ and R_2_, *θ_N2_* and *θ_H_* are the coverages of N_2_ and H, ${c^{'}}_{+}$ and ${c^{'}}_{-}$are the concentrations of protons and electrons near the catalyst surface. According to the equation (1) and (2), increase of *θ_N2_* and decrease of *θ_H_* at meanwhile are effective to boost the yield rate of N_2_ formation and further improve N_2_ reduction selectivity to NH_3_.


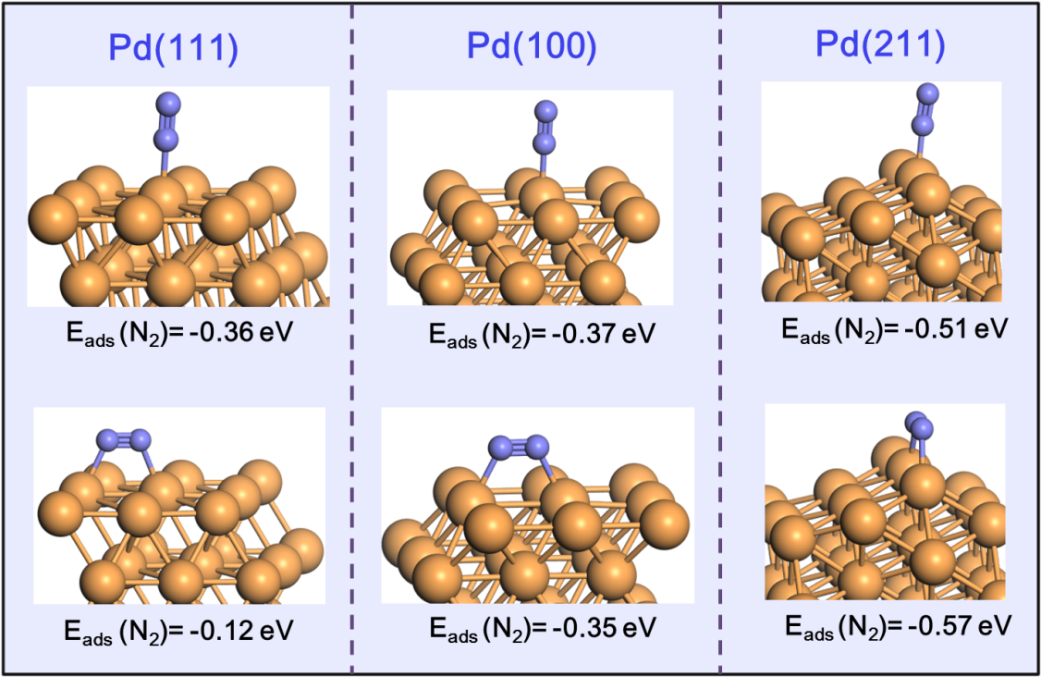


Figure S1: DFT calculations of E_ads_(N_2_) on Pd(111),(100) and (211) facets with vertical and parallel adsorption configuration of N_2_.


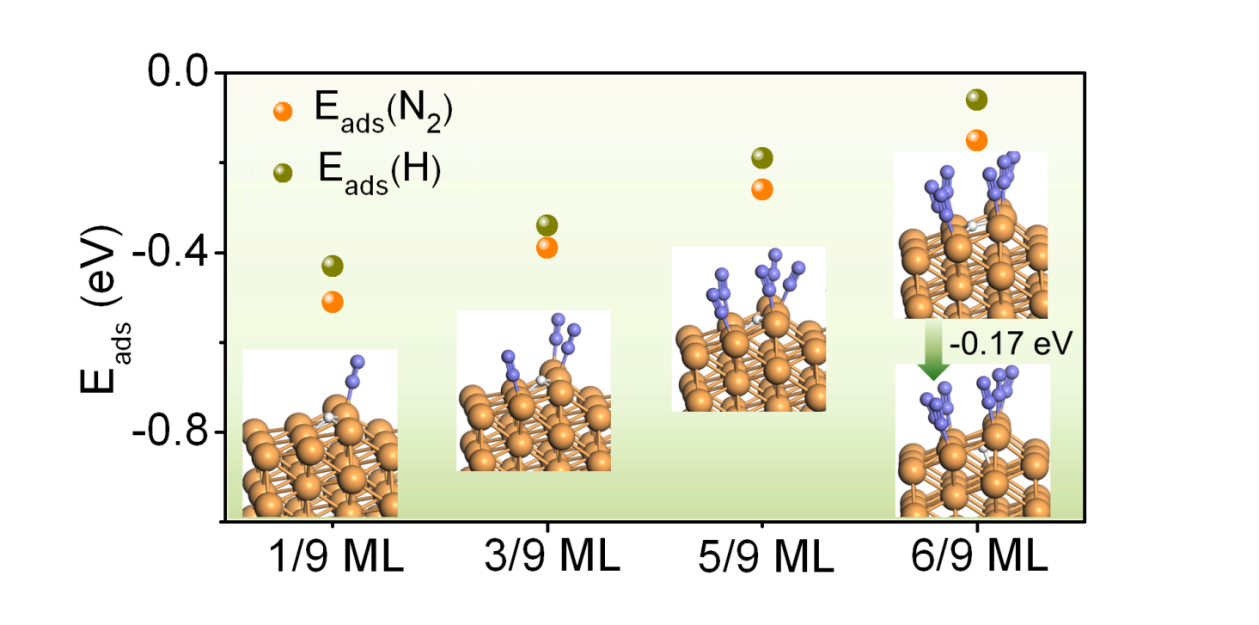


Figure S2: DFT calculations on the effect of *θ_N2_* on E_ads_(H) on the Pd(211) surface. White and purple balls denote H and N, respectively.


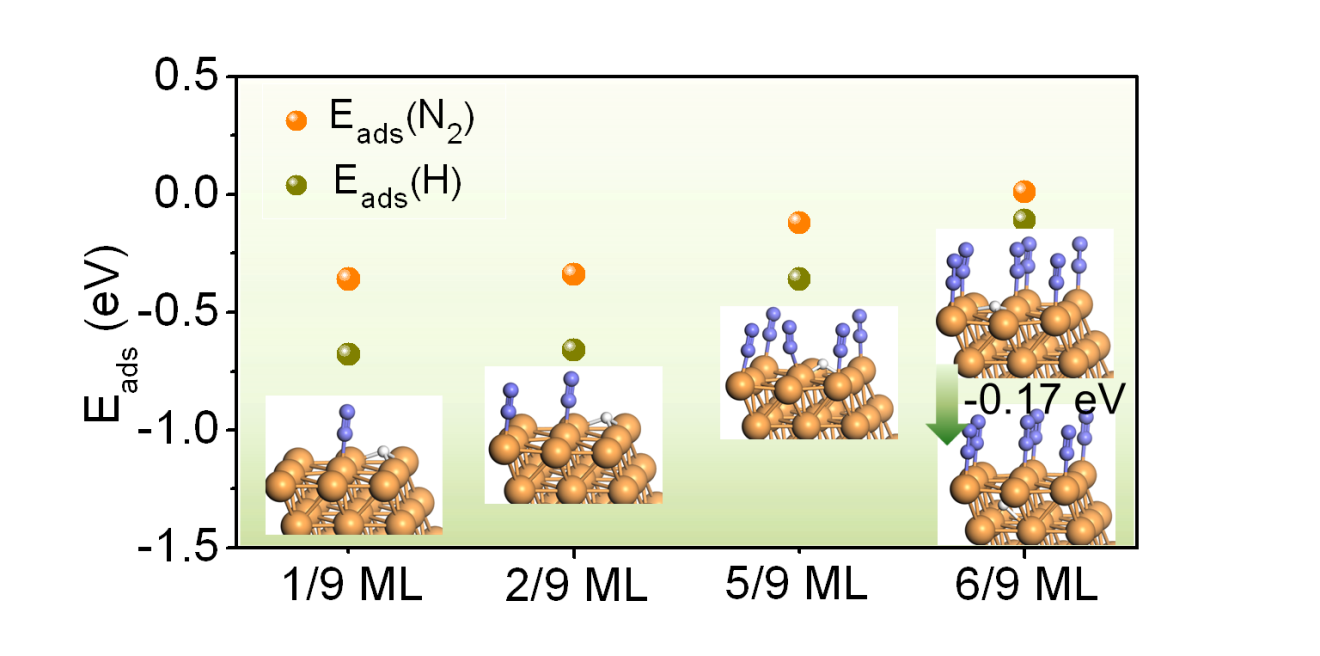


Figure S3: DFT calculations on the effect of*θ_N2_* on E_ads_(H) on the Pd(111) surface. White and purple balls denote H and N, respectively.


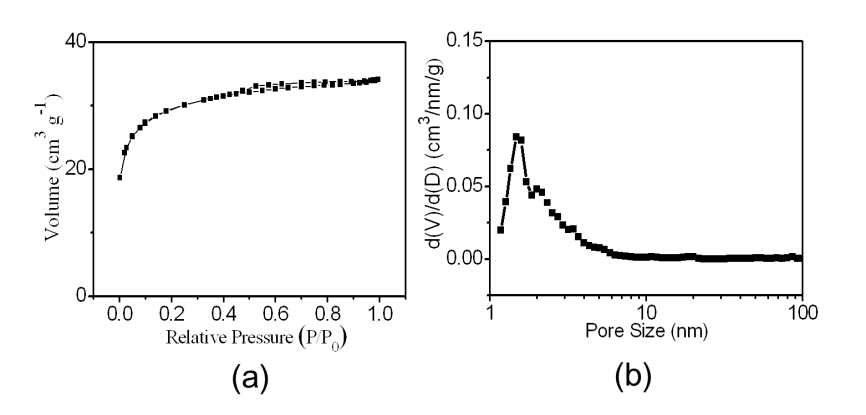


Figure S4: N_2_ adsorption and desorption analysis of ACC. (a) N_2_ adsorption and desorption curve and (b) pore size distribution of ACC.


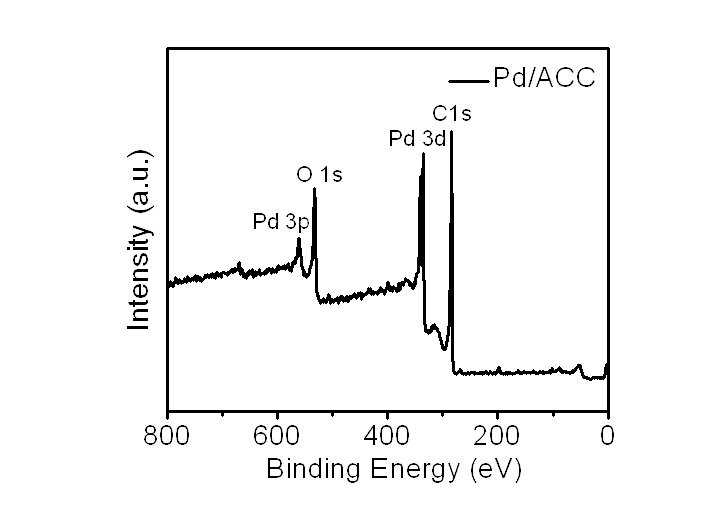


Figure S5: XPS spectrum of Pd/ACC.


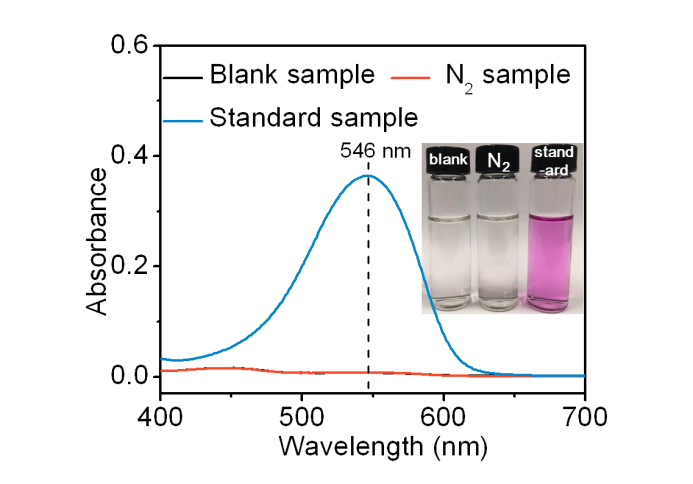


Figure S6: UV-vis adsorption spectrum of nitrite detection. Feeding ultra-high purity N_2_ into electrolytic cell for 2 h, with comparisons of the blank sample and the standard NaNO_2_ sample and the inset is the photo of the samples.


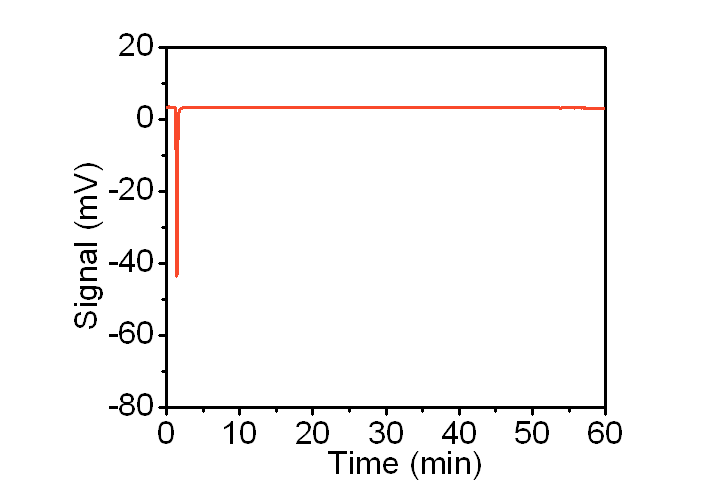


Figure S7: Gas chromatographic curve of ultra-high purity N_2_.


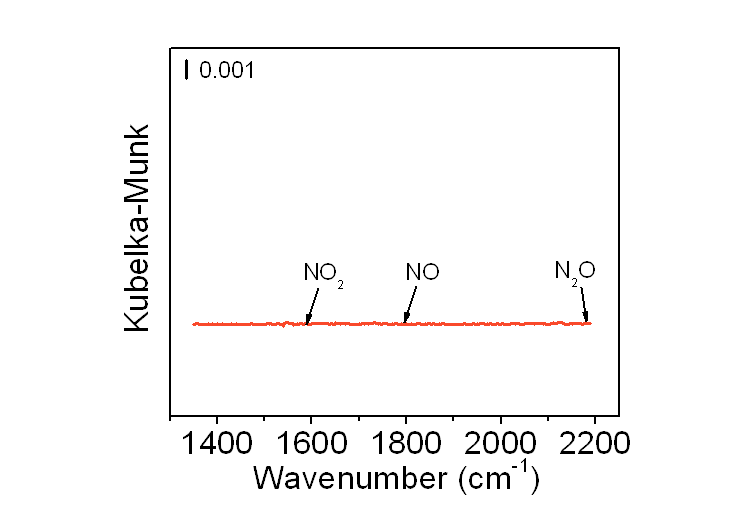


Figure S8: DRIFT spectrum of ultra-high purity N_2_ by subtracting the signal of Ar background.


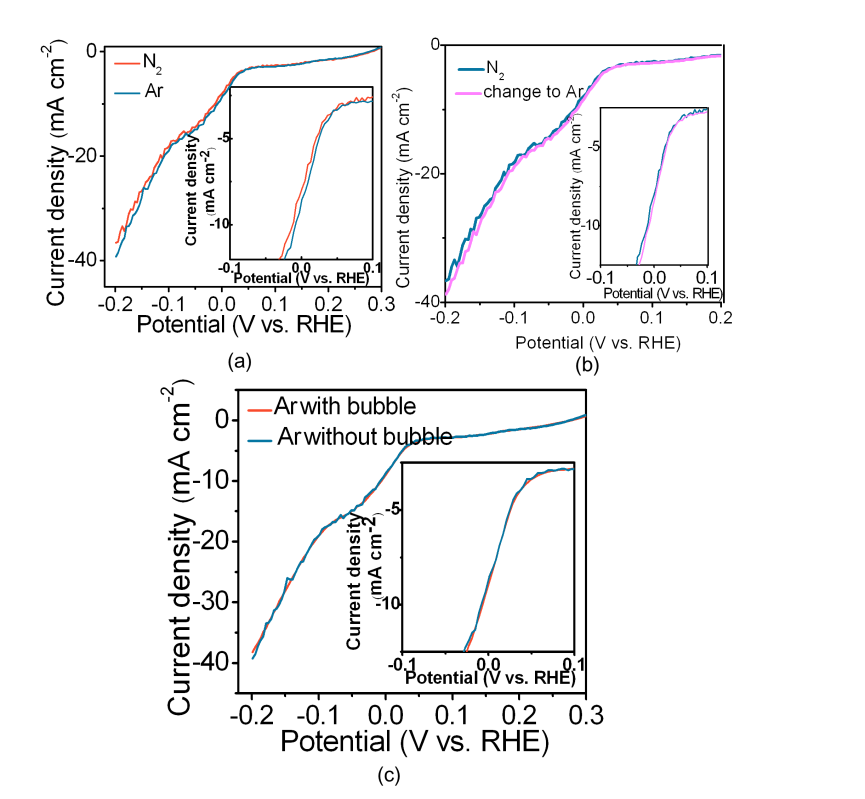


Figure S9: LSV curves of Pd/ACC under Ar and N_2_ condition. (a) LSV curve of Pd/ACC in N_2_ or Ar saturated 0.1 M HCl with bubbles covering. (b) LSV curves of Pd/ACC in N_2_ saturated 0.1 M HCl solution with bubbles covering on the catalyst, compared with that once N_2_ was changed to Ar. (c) LSV curve of Pd/ACC in Ar saturated 0.1 M HCl with or without bubbles covering. These phenomena were repeated. These phenomena were repeated and demonstrated that the decreased current density was because of the suppressed HER activity when N_2_ was covered on the surface of the Pd/ACC catalyst.


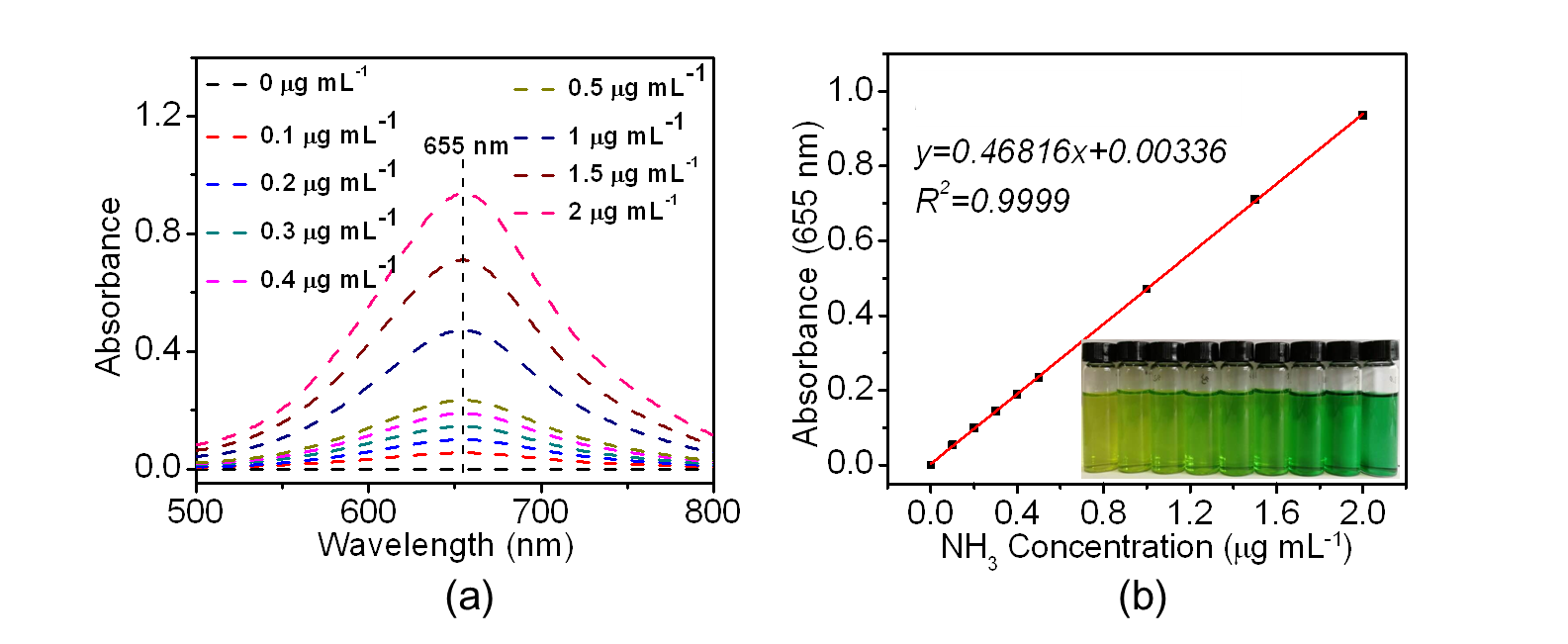


Figure S10: Calibration of the indophenol blue method using standard ammonia chloride solutions with a series of concentrations. (a) UV-vis curves (the background value for blank solution without NH_4_^+^ was subtracted from all the data) and (b) Concentration- Absorbance of NH_4_^+^ ions. The absorbance of 655 nm was measured by UV-vis spectrophotometer. The standard curve showed good linear relation of absorbance with NH_4_^+^ concentration (y=0.46816x+0.0036, R^2^=0.9999).


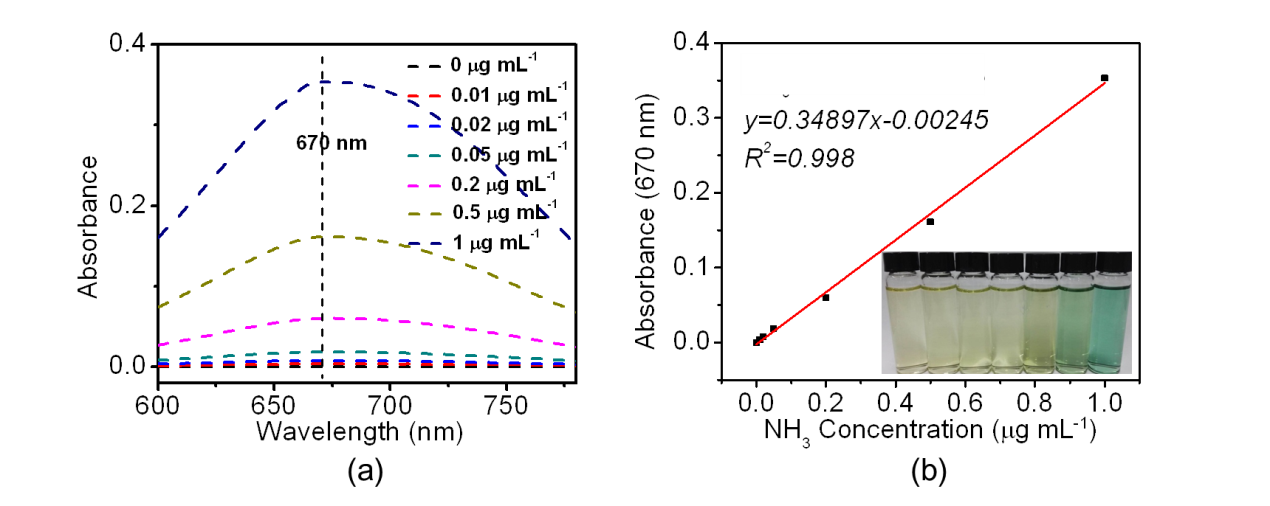


Figure S11: Calibration curve of the salicylic acid method using standard ammonia chloride solutions with a series of concentrations. (a) UV-vis curves (the background value for blank solution without NH_4_^+^ was subtracted from all the data) and (b) Concentration- Absorbance of NH_4_^+^ ions. The absorbance of 670 nm was measured by UV-vis spectrophotometer. The standard curve showed good linear relation of absorbance with NH_4_^+^ concentration (y=0.34897x-0.00245, R^2^=0.998).


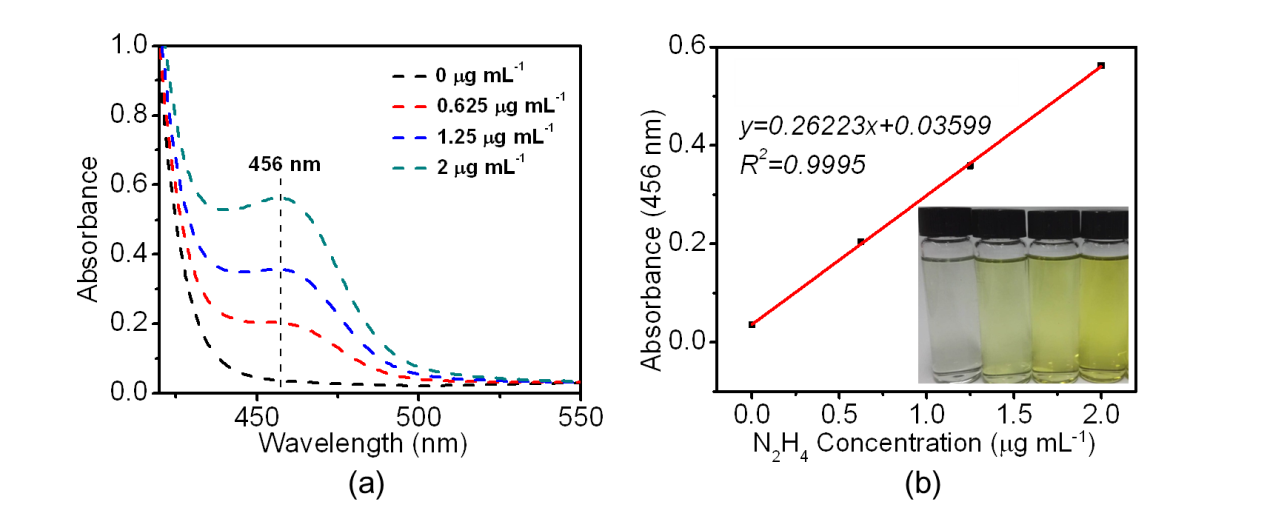


Figure S12: Calibration curve for N_2_H_4_ using standard N_2_H_4_ solutions with a series of concentrations. (a) UV-vis curves and (b) concentration-absorbance of N_2_H_4_. The absorbance of 456 nm was measured by UV-vis spectrophotometer. The standard curve showed good linear relation of absorbance with N_2_H_4_ concentration (y=0.26223x+0.03599, R^2^=0.992).


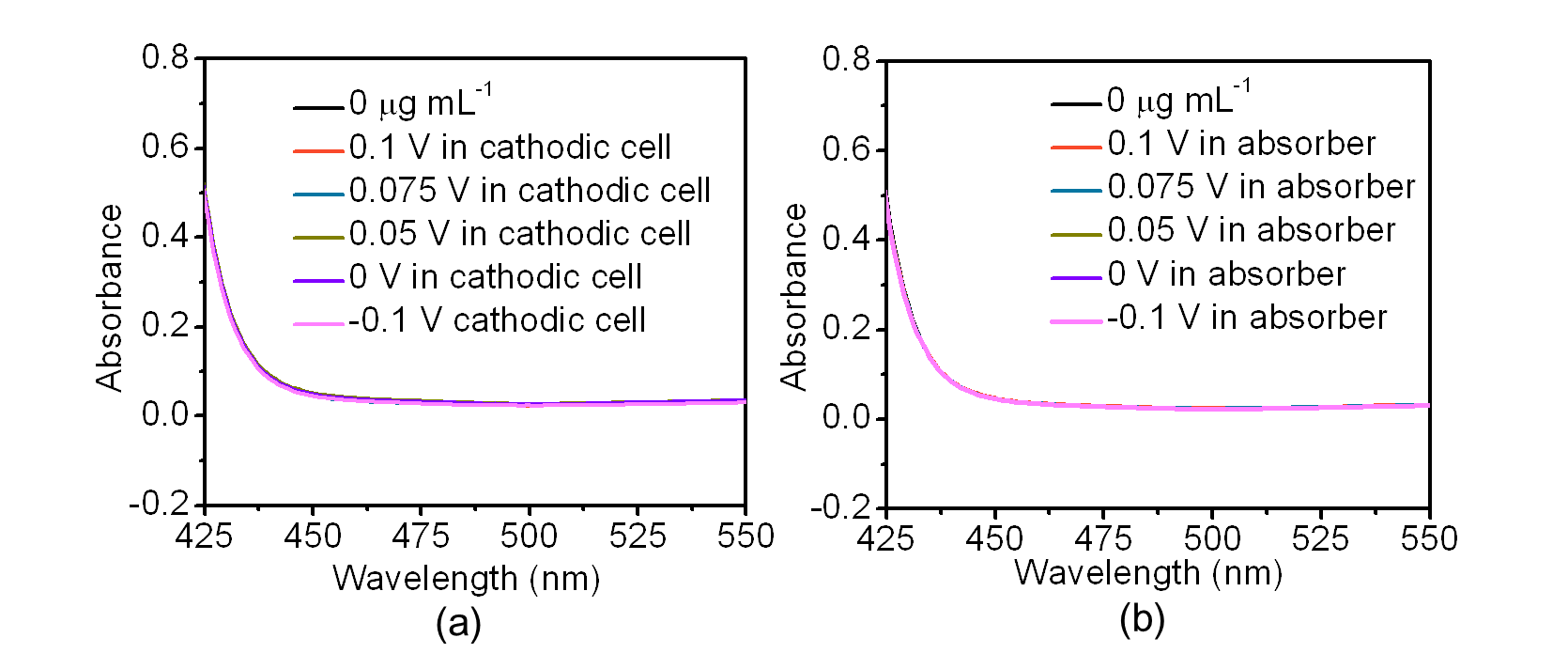


Figure S13: UV-vis adsorption spectra of N_2_H_4_ detection in HCl solution after 2 h electrolysis. (a) cathodic cell and (b) gas absorber under different potentials with the method of Watt and Chrisp.


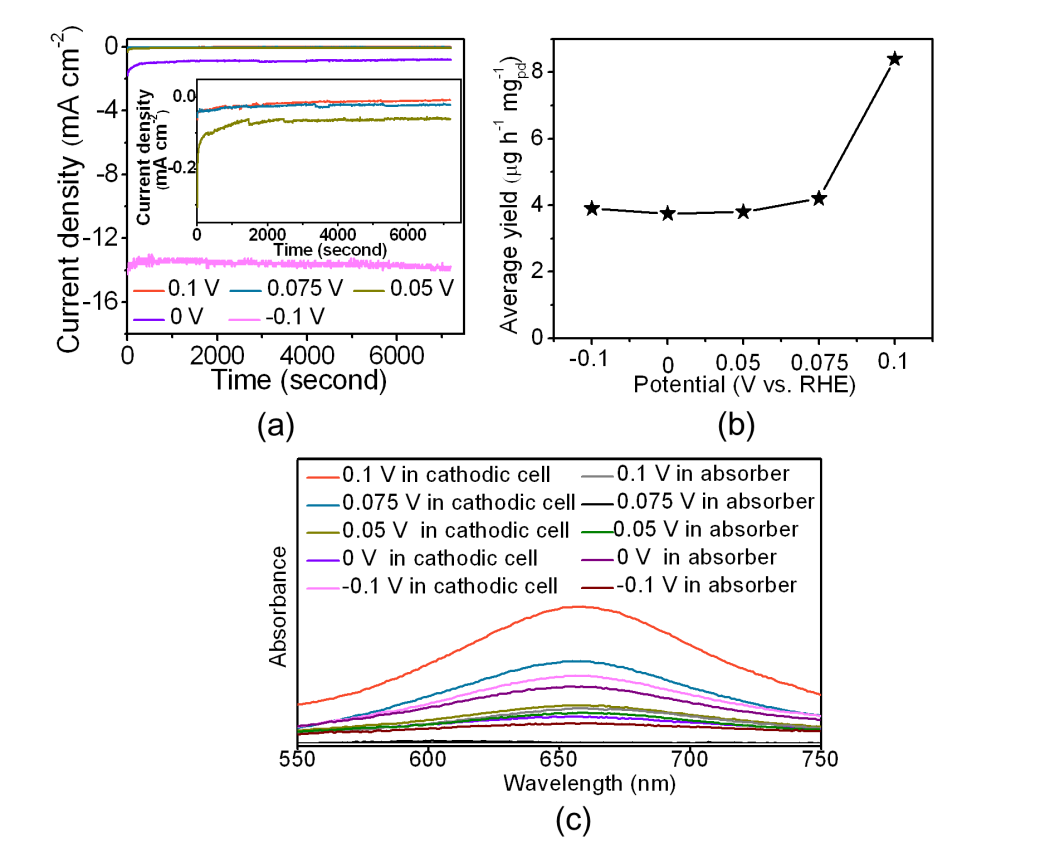


Figure S14: Chrono-amperometry curves and average yield rate of NH_3_ production of Pd/ACC at various potentials for 2 h. (a) Chrono-amperometry curves of Pd/ACC at various potentials for 2 h. (b) Average yield rate. (c) UV-vis adsorption spectra of indophenol blue method (the values were corrected by subtracting blank control).


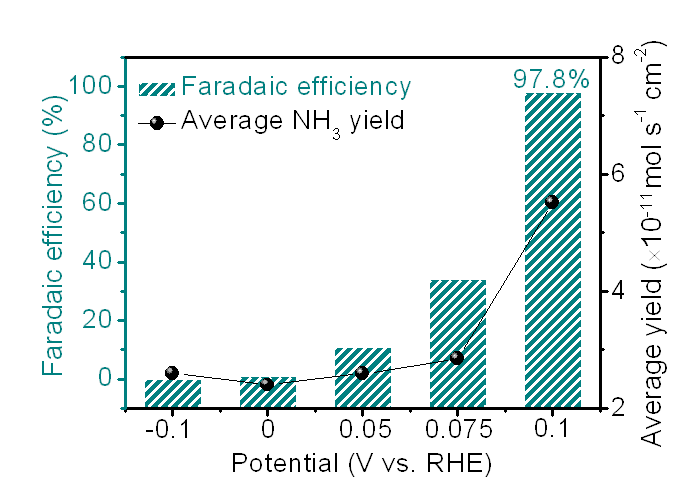


Figure S15**:** Faradaic efficiency and yield rate of NH_3_ production on Pd/ACC based on salicylic acid method.


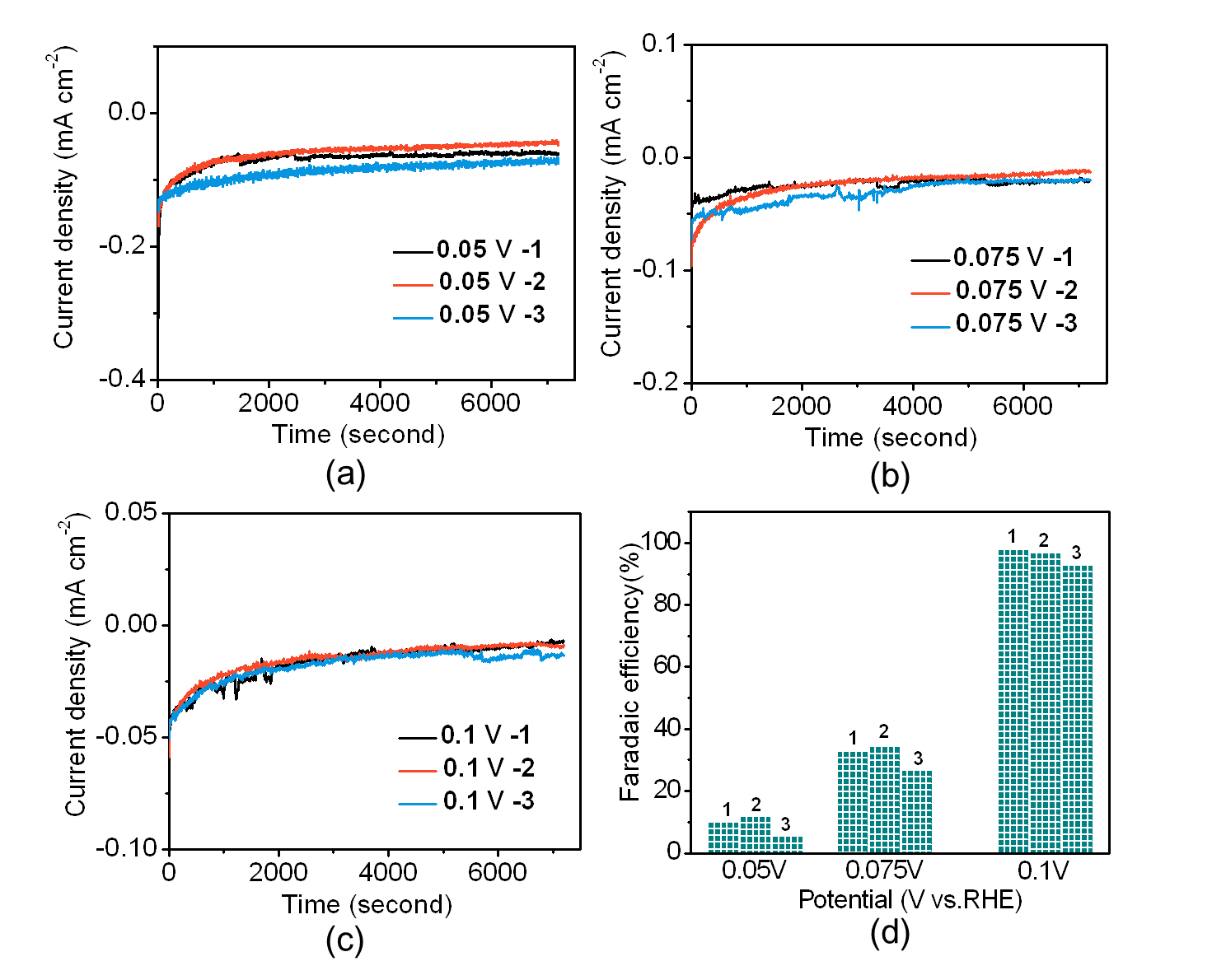


Figure S16：Repeated tests of Pd/ACC for three times. Repeated chrono-amperometry results of Pd/ACC catalyst in N_2_-saturated HCl with a flow rate of 130 sccm at (a) 0.05 V, (b) 0.075 V and (c) 0.1 V for 2 h for three times and (d) the corresponding FEs for the repeated tests at 0.05 V, 0.075 V and 0.1 V.


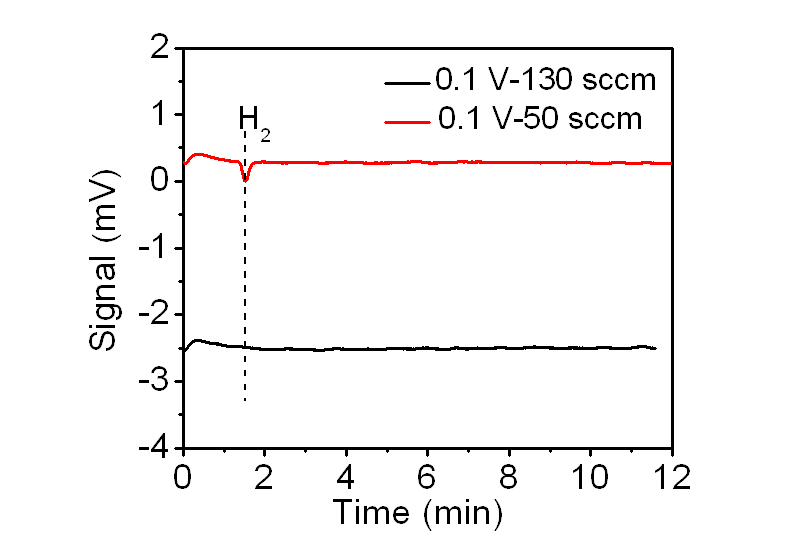


Figure S17: Gas chromatographic curves (Fuli 9790) for H_2_ detection during the NRR test at 0.1 V under 130 and 50 sccm of N_2_. N_2_ was used as a carrier gas.


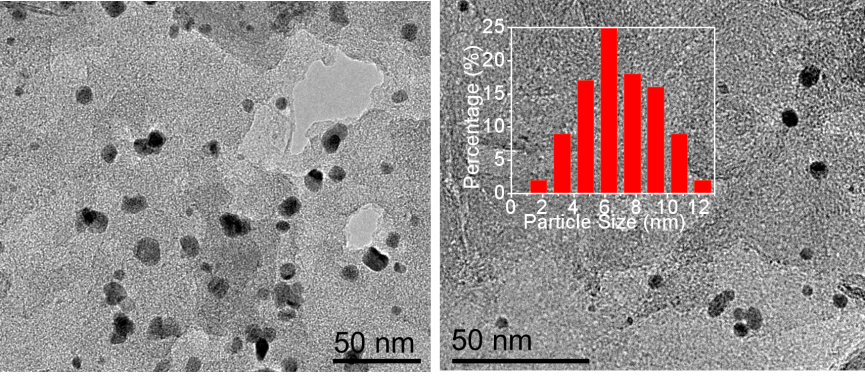


Figure S18: HRTEM images of Pd/ACC after N_2_ electrochemical reduction. The size distribution of Pd nanoparticles was shown in the inset.


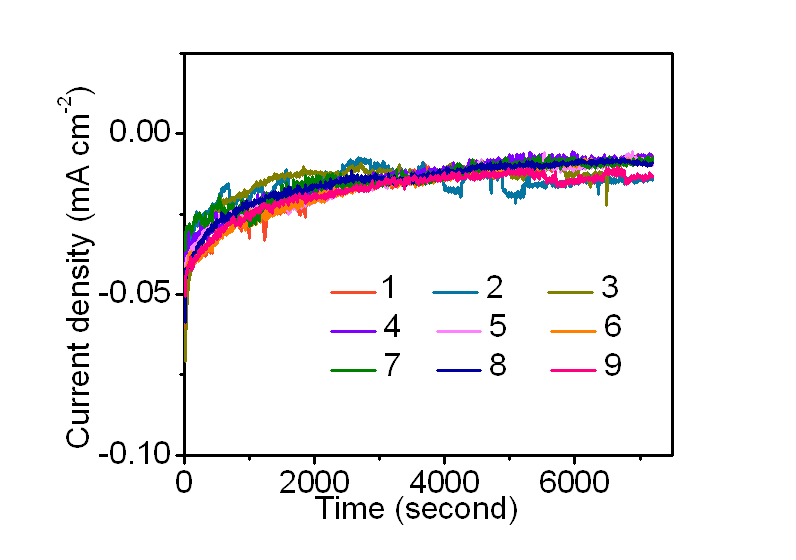


Figure S19: Chrono-amperometry results of Pd/ACC in N_2_-saturated HCl at 0.1 V vs RHE for cycles.


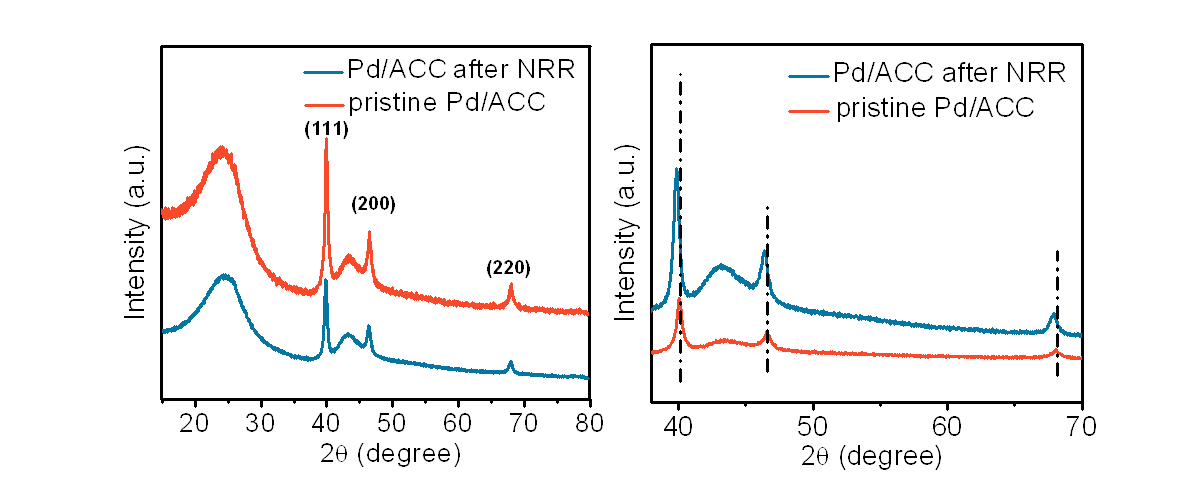


Figure S20: XRD spectra of Pd/ACC before and after NRR test at 0.1V.


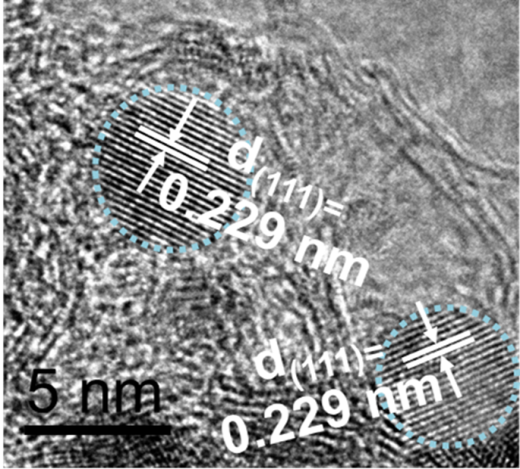


Figure S21: HRTEM image of Pd/ACC after NRR test at 0.1V.


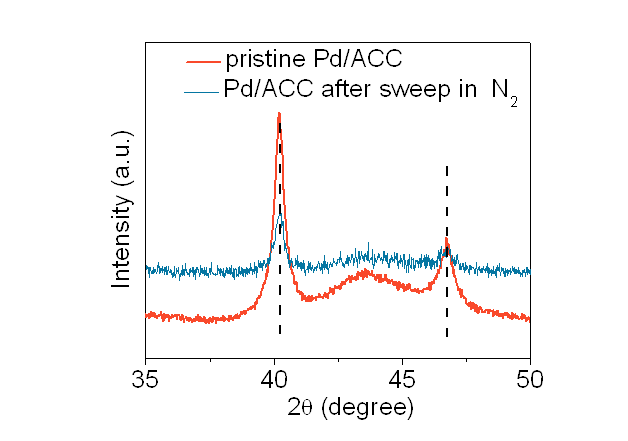


Figure S22: XRD image of Pd/ACC after heating in N_2_ in a tube [furnace](javascript:void(0);), compared with that of the pristine Pd/ACC.


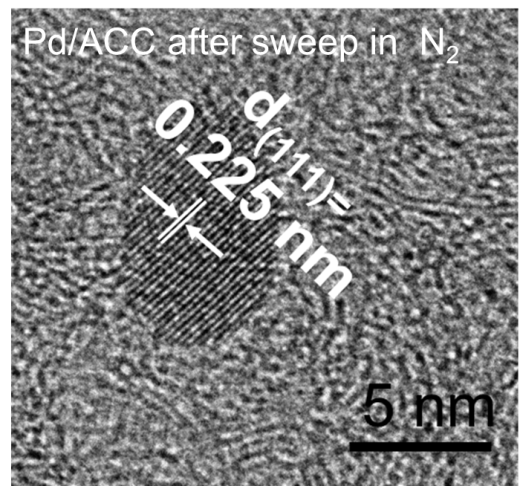


Figure S23: HRTEM image of Pd/ACC after sweep in N_2_ atmosphere in a tube [furnace](javascript:void(0);).


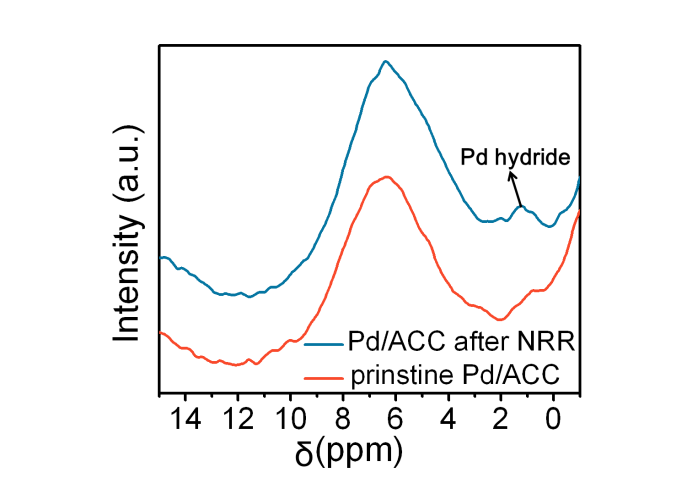


Figure S24: Solid-state ^1^H NMR spectra of Pd/ACC before and after NRR test at 0.1V [[3](#_ENREF_3)].


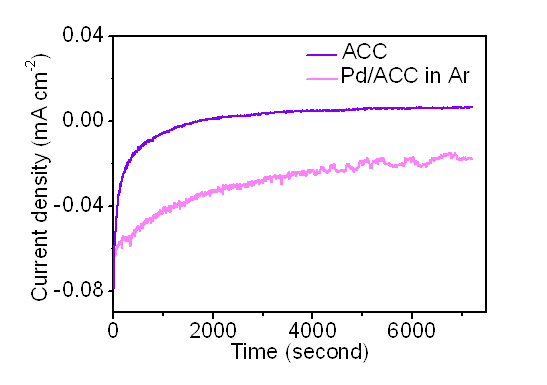


Figure S25**:** Chrono-amperometry results of ACC in N_2_-saturated HCl and Pd/ACC in Ar-saturated HCl at 0.1 V.


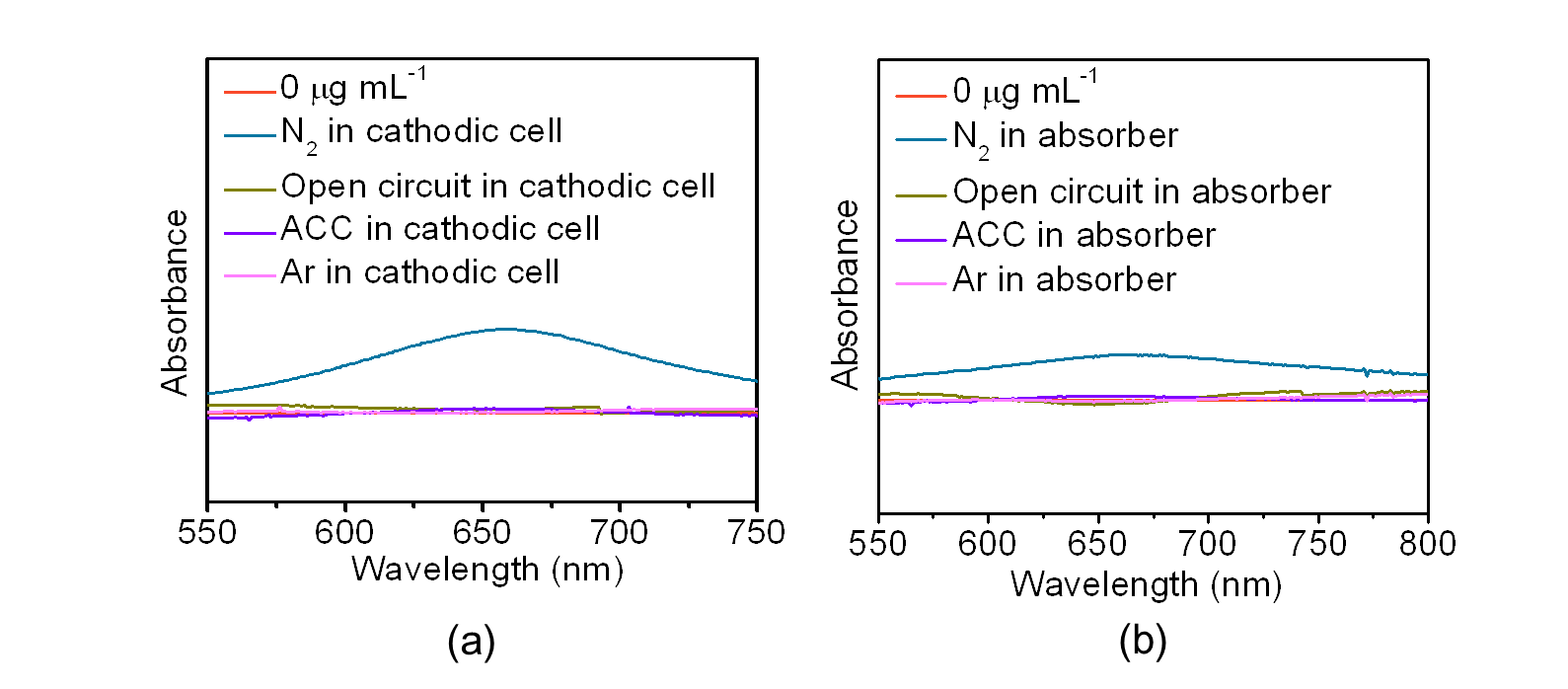


Figure S26: UV-vis adsorption spectra of NH_4_^+^ detection in gas absorber after 2 h electrolysis of contrast samples. UV-vis adsorption spectra of NH_4_^+^ detection in (a) cathodic cell and (b) gas absorber after 2 h electrolysis of contrast samples. Open circuit, ACC as the catalyst and Ar-saturated HCl solution were compared with that for Pd/ACC at 0.1 V in N_2_ by using indophenol blue method. The values were corrected by subtracting blank control. Open circuit represented that Pd/ACC was conducted in N_2_-saturated HCl for 2 h at open circuit. ACC represented that ACC was conducted in N_2_-saturated HCl for 2 h at 0.1 V. Ar represented that Pd/ACC was conducted in Ar-saturated HCl for 2 h at 0.1 V.


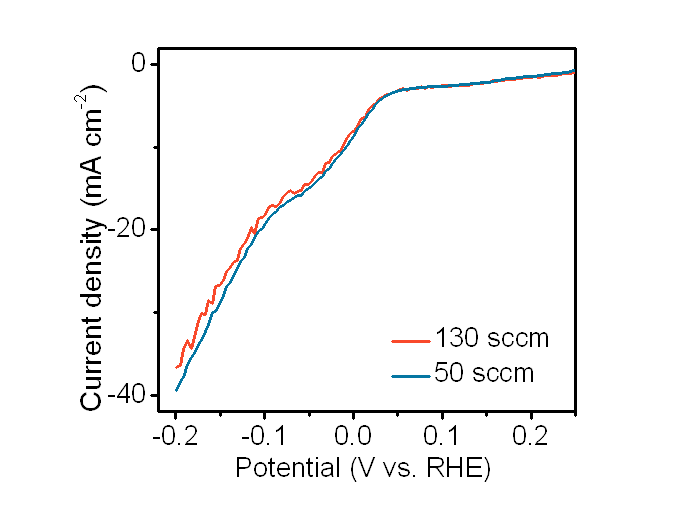


Figure S27: LSV curves in N_2_ saturated HCl at a gas flow rate of 50 sccm and 130 sccm with bubbles covering on the catalyst.


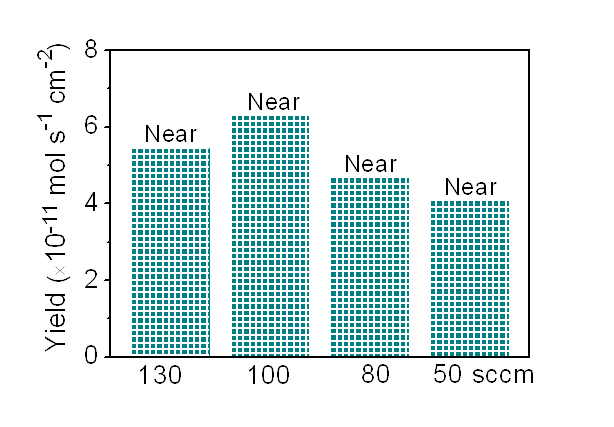


Figure S28: Average ammonia yield rates for Pd/ACC at 0.1 V under various flow rates of N_2_.


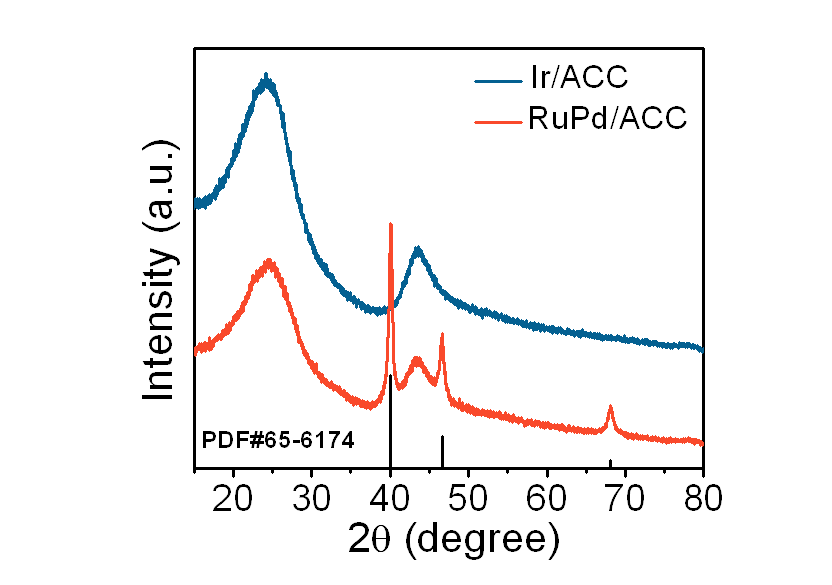


Figure S29: XRD patterns of Ir/ACC and RuPd/ACC.


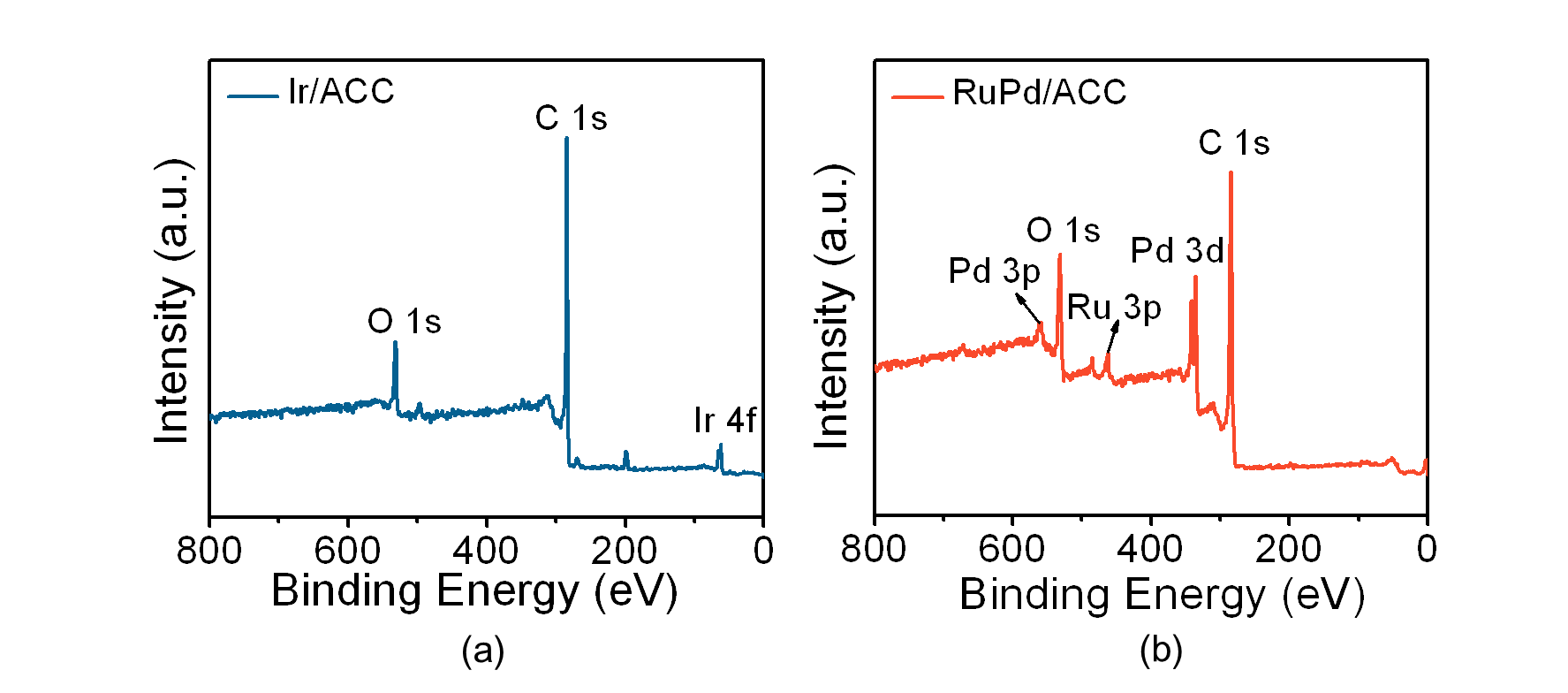


Figure S30: XPS spectra. (a) Ir/ACC and (b) RuPd/ACC, indicating Ir-based and RuPd-based catalysts were successfully prepared.


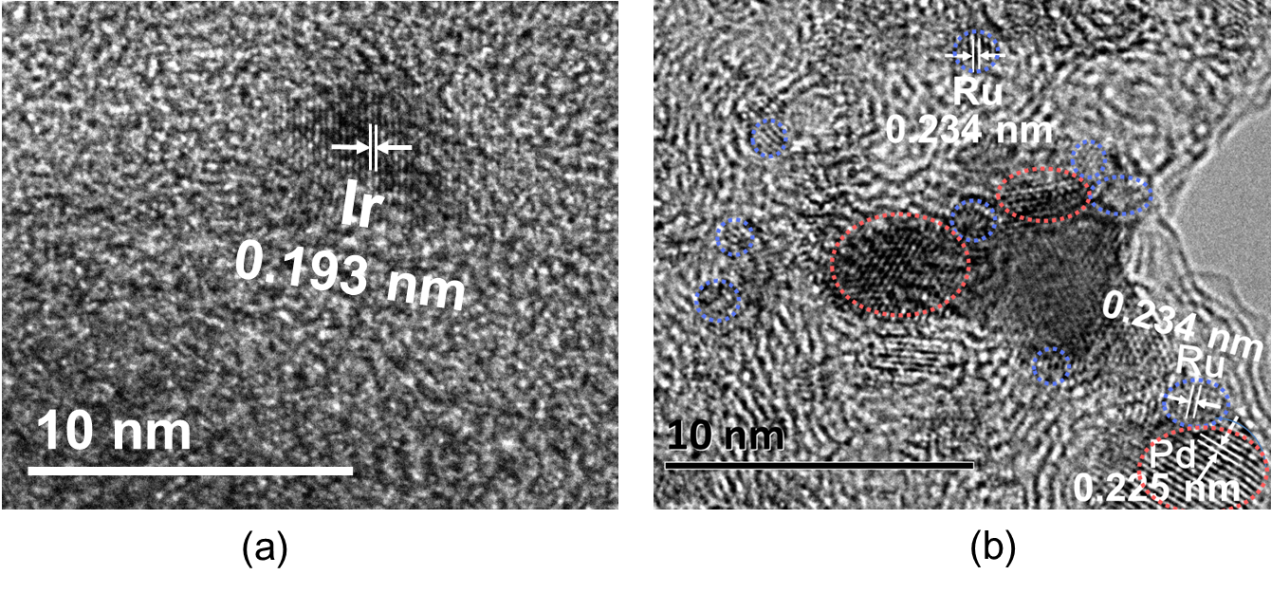


Figure S31: HRTEM images. (a) Ir/ACC and (b) RuPd/ACC.


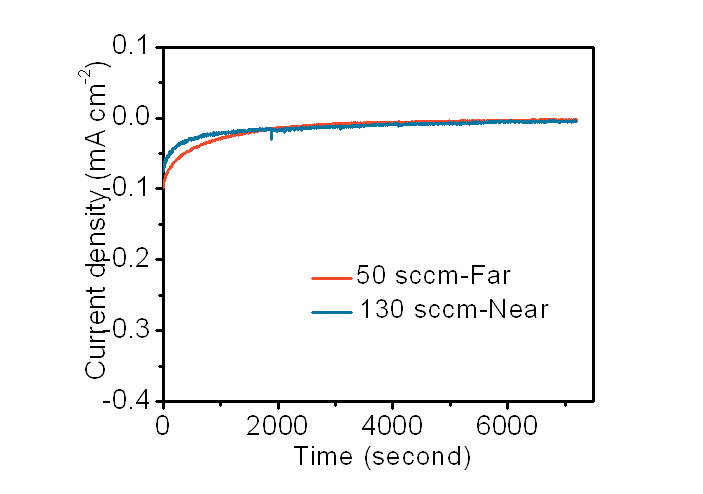


Figure S32: Chrono-amperometry results of Ir/ACC (N_2_-saturated HCl at 0.1 V under a N_2_ flow rate of 130 sccm with gas tube near the electrode to ensure N_2_ bubbles surrounding the catalyst, with a comparison of that under a N_2_ flow rate of 50 sccm with gas tube far from the electrode to ensure less N_2_ bubble hitting the catalyst).


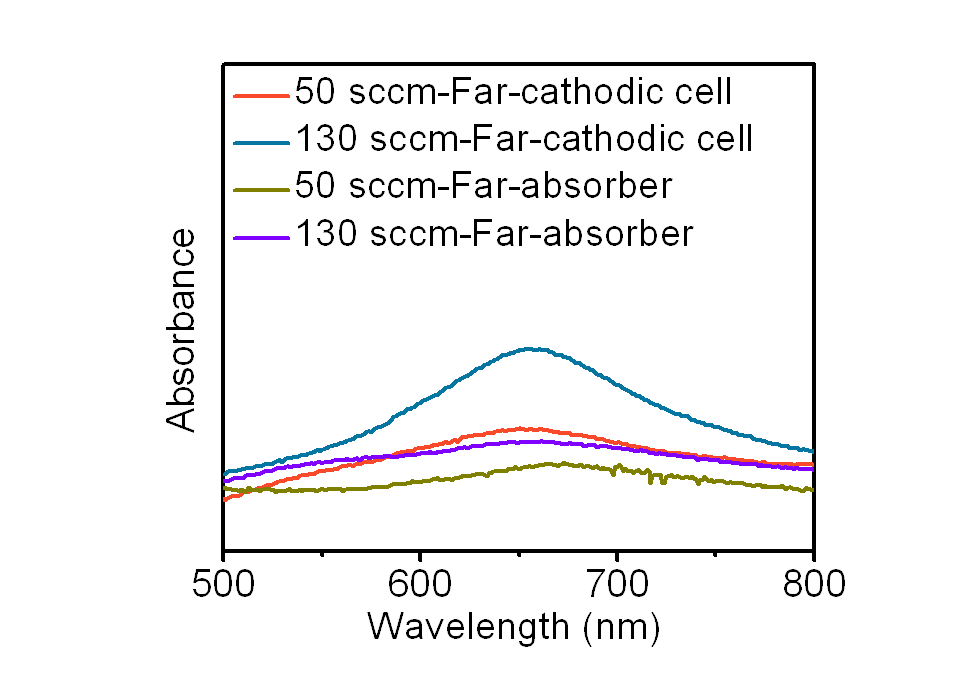


Figure S33: UV-vis adsorption spectra of NH_4_^+^ detection of Ir/ACC catalyst. UV-vis adsorption spectra (the values were corrected by subtracting blank control) of NH_4_^+^ detection after 2 h electrolysis of Ir/ACC catalyst at 0.1 V under a N_2_ flow rate of 130 sccm with gas tube near the electrode and a N_2_ flow rate of 50 sccm with gas tube far from the electrode.


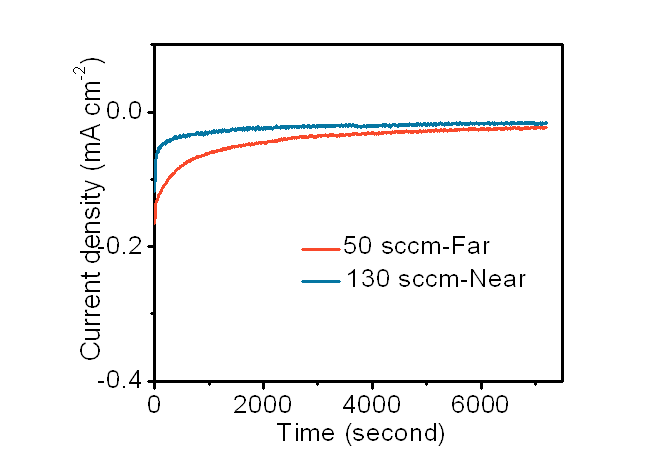


Figure S34: Chrono-amperometry results of RuPd/ACC. N_2_-saturated HCl at 0.1 V under a N_2_ flow rate of 130 sccm with gas tube near the electrode to ensure N_2_ bubbles surrounding the catalyst, with a comparison of that under a N_2_ flow rate of 50 sccm with gas tube far from the electrode to ensure less N_2_ bubble hitting the catalyst.


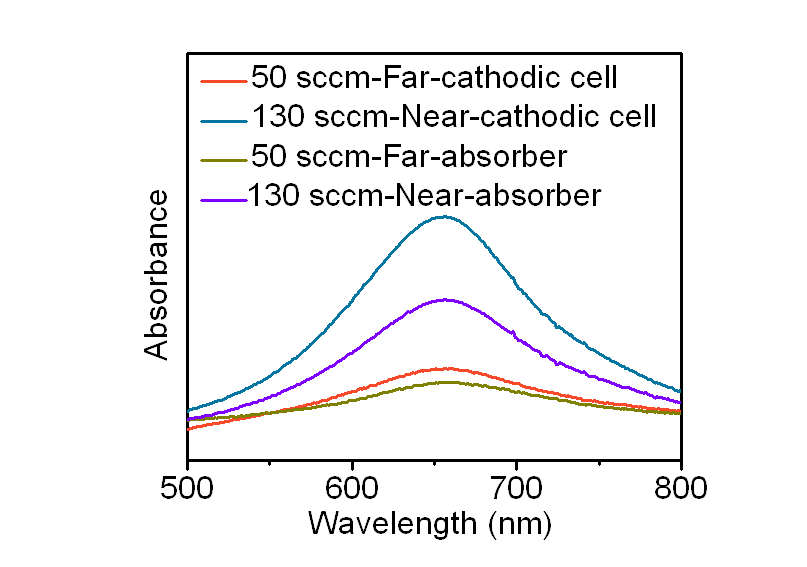


Figure S35: UV-vis adsorption spectra of NH_4_^+^ detection of RuPd/ACC catalyst. UV-vis adsorption spectra (the values were corrected by subtracting blank control) of NH_4_^+^ detection after 2 h electrolysis of RuPd/ACC catalyst at 0.1 V under a N_2_ flow rate of 130 sccm with gas tube near the electrode and a N_2_ flow rate of 50 sccm with gas tube far from the electrode (the values were corrected by subtracting blank control).

.


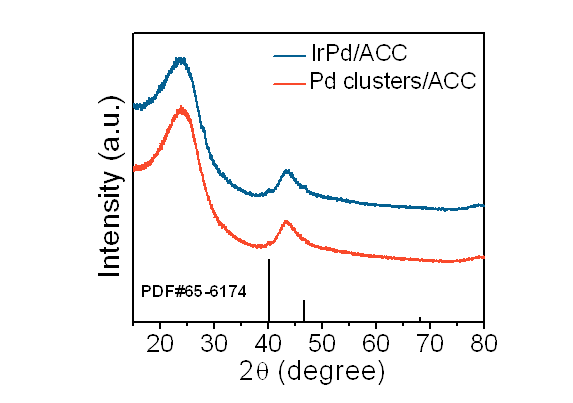


Figure S36: XRD patterns of IrPd/ACC and Pd clusters/ACC.


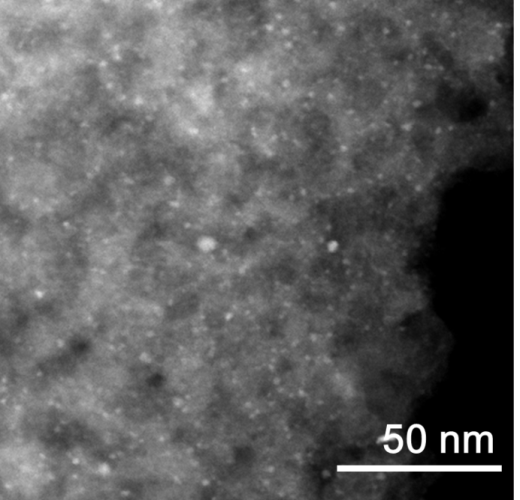


Figure S37: HAADF-STEM image of IrPd/ACC.


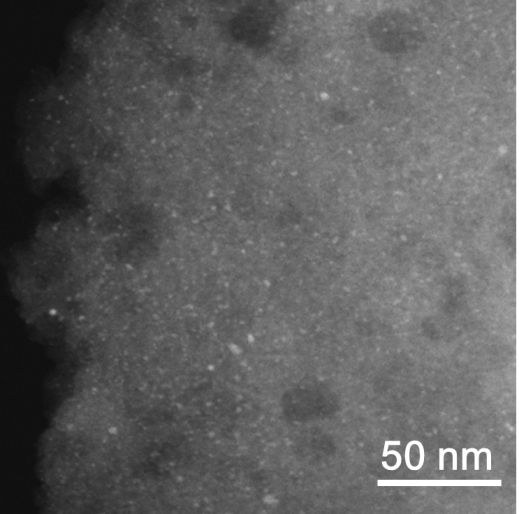


Figure S38: HAADF-STEM image of Pd clusters/ACC.


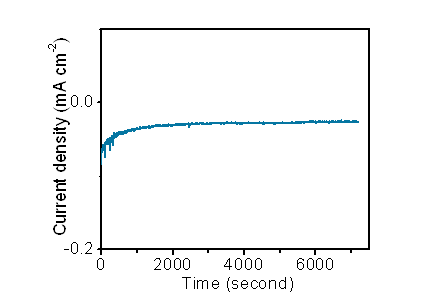


Figure S39: Chrono-amperometry result of IrPd/ACC. (N_2_-saturated HCl at 0.1 V under a N_2_ flow rate of 130 sccm with gas tube near the electrode to ensure N_2_ bubbles surrounding the catalyst).


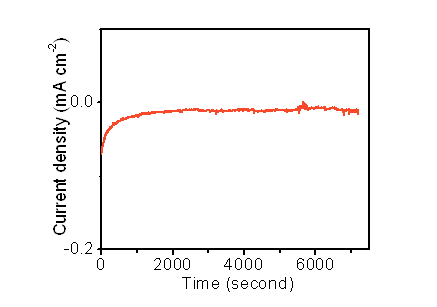


Figure S40: Chrono-amperometry result of Pd clusters/ACC. (N_2_-saturated HCl at 0.1 V under a N_2_ flow rate of 130 sccm with gas tube near the electrode to ensure N_2_ bubbles surrounding the catalyst).


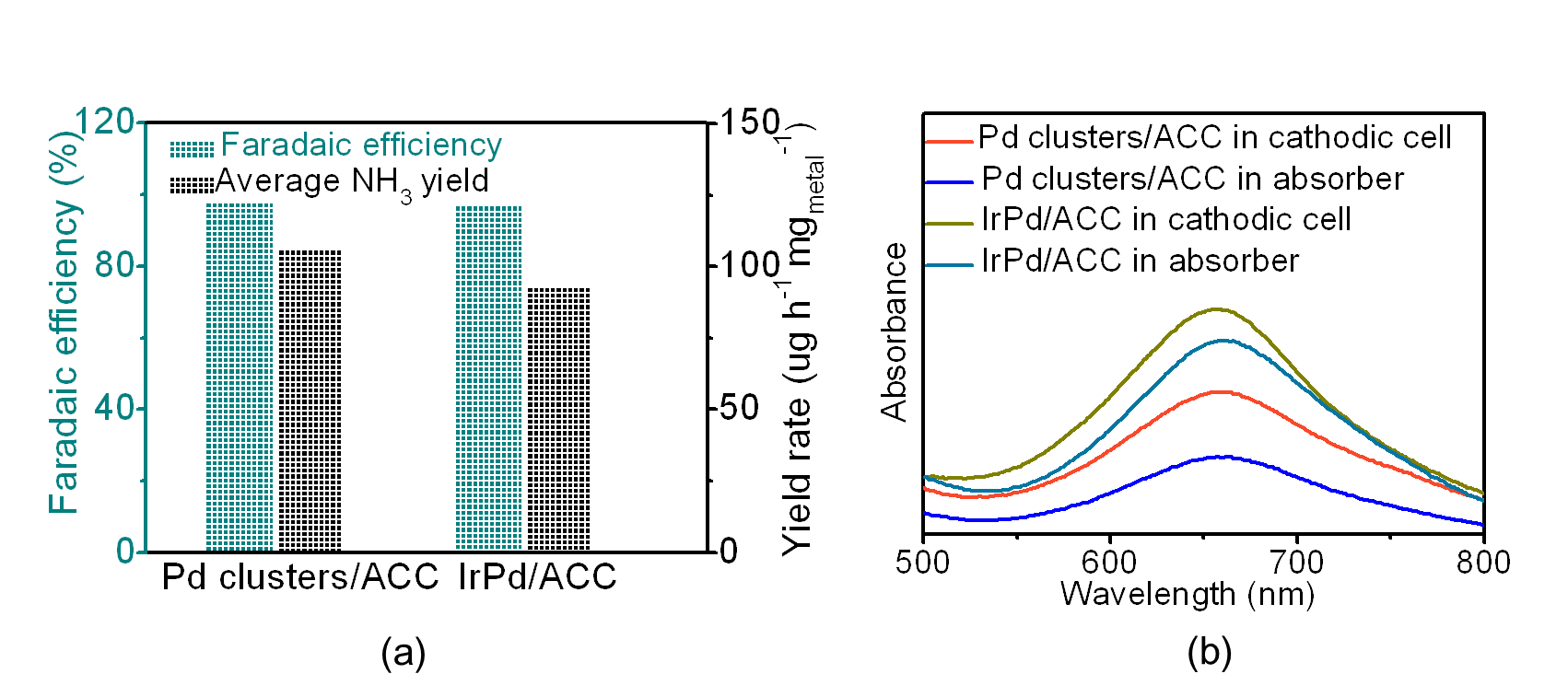


Figure S41: FEs and average NH_3_ yield rates of Pd clusters/ACC and IrPd/ACC at 0.1 V with the adjusted three-phase interface. (a) FEs and average NH_3_ yields. (b) UV-vis adsorption spectra (the values were corrected by subtracting blank control).

**Table S1.**

Comparison of the NRR performance of Pd/ACC with other catalysts recently reported under ambient conditions (room temperature and atmospheric pressure).

| Catalyst | Electrolyte | Faradaic efficiency (FE, %) |
| --- | --- | --- |
| **Pd/ACC, this work (130 sccm, Near)** | **0.1 M HCl** | **97** |
| Pd/ACC, this work (50 sccm, Far) | 0.1 M HCl | 26 |
| PdO/Pd/CNTs，Ref.[[4](#_ENREF_4)] | 0.1 M NaOH | 11.5 |
| AuPdP NWs，Ref.[[5](#_ENREF_5)] | 0.1 M Na_2_SO_4_ | 15.44 |
| PdRu BPNs，Ref.[[6](#_ENREF_6)] | 0.1 M HCl | 1.53 |
| Pd/carbon black, Ref. [[7](#_ENREF_7)] | 0.1 M PBS | 8 |
| Fe_SA_-N-C, Ref. [[8](#_ENREF_8)] | 0.1 M KOH | 57 |
| Au nanorods, Ref. [[9](#_ENREF_9)] | 0.1 M KOH | 4 |
| Bi_4_V_2_O_11_-CeO_2_, Ref. [[10](#_ENREF_10)] | 0.1 M HCl | 10 |
| Mo_2_C/C, Ref. [[11](#_ENREF_11)] | 0.5 M Li_2_SO_4_, pH=3 | 8 |
| Ru SA@ZrO_2_/NC, Ref. [[12](#_ENREF_12)] | 0.1 M HCl | 21 |
| BiNCs, Ref. [[13](#_ENREF_13)] | 0.5 M K_2_SO_4_, pH=3.5 | 67 |
| MXene, Ref. [[14](#_ENREF_14)] | HCl-Li_2_SO_4_, pH=2 | 6 |
| VN, Ref. [[15](#_ENREF_15)] | 1 mM H_2_SO_4_ | 6 |
| B-doped Graphene, Ref. [[16](#_ENREF_16)] | 0.05 M H_2_SO_4_ | 11 |

**Movie S1.**

Electrochemical NRR test with the adjusted three-phase interface.

**Supplemental Reference**

[1] D. Wang, L. M. Azofra, M. Harb *et al.*, "E Energy-efficient nitrogen reduction to ammonia at low overpotential in aqueous electrolyte under ambient conditions." *ChemSusChem*, vol. 11, no. 19, pp. 3416-3422, 2018.

[2] A. R. Singh, B. A. Rohr, J. A. Schwalbe *et al.*, " Electrochemical ammonia synthesis-the selectivity challenge." *ACS Catalysis*, vol. 7, no. 1, pp. 706-709, 2017.

[3] S. Dekura, H. Kobayashi, R. Ikeda *et al.*, " The electronic state of hydrogen in the phase of the hydrogen-storage material PdH(D)(x): Does a chemical bond between palladium andhydrogen exist?" *Angew. Chem., Int. Ed.*, vol. 57, no. 31, pp. 9823-9827, 2018.

[4] J. Lv, S. Wu, Z. Tian *et al.*, "Construction of PdO-Pd interfaces assisted by laser irradiation for enhanced electrocatalytic N_2_ reduction reaction." *J. Mater. Chem. A*, vol. 7, no. 20, pp. 12627-12634, 2019.

[5] H. Wang, D. Yang, S. Liu *et al.*, "Metal–nonmetal one-dimensional electrocatalyst: AuPdP nanowires for ambient nitrogen reduction to ammonia." *ACS Sustainable Chem. Eng.*, vol. 7, no. 18, pp. 15772-15777, 2019.

[6] Z. Wang, C. Li, K. Deng *et al.*, "Ambient nitrogen reduction to ammonia electrocatalyzed by bimetallic PdRu porous nanostructures." *ACS Sustainable Chem. Eng.*, vol. 7, no. 2, pp. 2400-2405, 2019.

[7] J. Wang, L. Yu, L. Hu *et al.*, " Ambient ammonia synthesis via palladium-catalyzed electrohydrogenation of dinitrogen at low overpotential." *Nat. Commun.*, vol. 9, Article ID 1795, 2018.

[8] M. Wang, S. Liu, T. Qian *et al.*, "Over 56.55% Faradaic efficiency of ambient ammonia synthesis enabled by positively shifting the reaction potential." *Nat. Commun.*, vol. 10, Article ID 341, 2019.

[9] M.-M. Shi, D. Bao, B.-R. Wulan *et al.*, " Au sub-nanoclusters on TiO_2_ toward highly efficient and selective electrocatalyst for N_2_ conversion to NH_3_ at ambient conditions." *Adv. Mater.*, vol. 29, no. 17, Article ID 1606550, 2017.

[10] J. Mao, W. Chen, W. Sun *et al.*, "Rational control of the selectivity of a ruthenium catalyst for hydrogenation of 4-nitrostyrene by strain regulation." *Angew. Chem., Int. Ed.*, vol. 56, no. 39, pp. 11971-11975, 2017.

[11] H. Cheng, L.-X. Ding, G.-F. Chen *et al.*, " Molybdenum carbide nanodots enable efficient electrocatalytic nitrogen fixation under ambient conditions." *Adv. Mater.*, vol. 30, no. 46, Article ID 1803694, 2018.

[12] H. Tao, C. Choi, L.-X. Ding *et al.*, " Nitrogen fixation by Ru single-atom electrocatalytic reduction." *Chem*, vol. 5, no. 1, pp. 204-214, 2019.

[13] Y. Hao, Y. Guo, L. Chen *et al.*, "Promoting nitrogen electroreduction to ammonia with bismuth nanocrystals and potassium cations in water." *Nat. Catal.*, 10.1038/s41929-019-0241-7, 2019.

[14] Y. Luo, G.-F. Chen, L. Ding *et al.*, " Efficient electrocatalytic N_2_ fixation with MXene under ambient conditions." *Joule*, vol. 3, no. 1, pp. 279-289, 2019.

[15] X. Yang, J. Nash, J. Anibal *et al.*, " Mechanistic insights into electrochemical nitrogen reduction reaction on vanadium nitride nanoparticles." *J. Am. Chem. Soc.*, vol. 140, no. 41, pp. 13387-13391, 2018.

[16] X. Yu, P. Han, Z. Wei *et al.*, " Boron-doped graphene for electrocatalytic N_2_ reduction." *Joule*, vol. 2, no. 8, pp. 1610-1622, 2018.
